# Epitope-directed selection of GPCR nanobody ligands with evolvable function

**DOI:** 10.1073/pnas.2423931122

**Published:** 2025-03-11

**Authors:** Meredith A. Skiba, Clare Canavan, Genevieve R. Nemeth, Jinghan Liu, Ali Kanso, Andrew C. Kruse

**Affiliations:** ^a^Department of Biological Chemistry and Molecular Pharmacology, Blavatnik Institute, Harvard Medical School, Boston, MA 02115

**Keywords:** GPCR, nanobody, angiotensin, allostery, cryo-EM

## Abstract

Antibodies have the potential to target G protein–coupled receptors (GPCRs) with high receptor, cellular, and tissue selectivity; however, few antibody ligands for GPCRs exist. Here, we describe a generalizable selection method to enrich for GPCR ligands from a synthetic camelid antibody fragment (nanobody) library. Our strategy yielded multiple nanobody ligands for the angiotensin II type I receptor (AT1R), a prototypical GPCR and important drug target. We found that nanobodies readily act as allosteric modulators, encoding selectivity for both the receptor and chemical features of GPCR ligands. We then used structure-guided design to convert two nanobodies from allosteric ligands to competitive AT1R inhibitors through simple mutations. This work demonstrates that nanobodies can encode multiple pharmacological behaviors and have great potential as evolvable scaffolds for the development of next-generation GPCR therapeutics.

## Significance

G protein–coupled receptors (GPCRs) regulate nearly all aspects of human physiology and are frequently drugged to treat cardiac, neurological, and metabolic diseases. Unlike other therapeutic targets, GPCRs are almost exclusively modulated by small-molecule ligands, as developing GPCR-targeting antibodies has been extremely challenging. We developed a generalizable strategy to identify antibody ligands for GPCRs and characterized how our antibody ligands modulate GPCR signaling. We then demonstrated that we can rapidly change the pharmacological behavior of our antibodies through simple mutagenesis. Our work shows that antibodies are effective GPCR ligands and could drug GPCRs that are not well targeted by small molecules with high specificity.

G protein–coupled receptors (GPCRs) sense hormonal, metabolic, and environmental cues and transmit this information across the cell membrane to initiate a broad range of cellular responses. Given their central role in human physiology, GPCRs are one of the most successful therapeutic targets for small-molecule and peptide drugs (1). Still, there is a push to develop molecules that target GPCRs with increased selectivity to reduce undesirable side effects. Antibodies have the potential to target GPCRs with higher precision, but the development of antibody-based therapeutics for GPCRs has lagged compared to other therapeutic targets due to limited quantities of stable, high-quality antigen and the high sequence conservation of GPCRs between species, which can hinder traditional immunization-based antibody discovery (2). Additionally, prototypical GPCRs have relatively small extracellular regions, leaving few accessible epitopes for recognition by conventional antibodies.

Camelid antibodies (nanobodies) have emerged as effective scaffolds that readily bind GPCRs and, in some cases, modulate their function (3–17). Due to their small size and long complementarity-determining region (CDR) 3, nanobodies are well poised to access the small solvent-accessible cavities on GPCR surfaces, which are inaccessible to conventional antibodies, where the antigen recognition motifs are spread across two chains (18). Additionally, nanobodies are amenable to combinatorial display technologies, which can be combined with synthetic libraries to overcome the challenges associated with protein stability and immunological tolerance across species (19–23). Even with these technological advances, the discovery of pharmacologically active nanobodies remains challenging.

We recently employed a synthetic library to discover nanobody ligands for the angiotensin II type I receptor (AT1R), a prototypical GPCR and important therapeutic target for the treatment of hypertension and kidney disease (8, 15). These nanobodies were generated via a ligand competition-based selection, which enriched for nanobodies that displace low-affinity ligands from AT1R’s orthosteric pocket and act as competitive and allosteric modulators (24). Here, we sought to develop a more universal approach to identify nanobody ligands for GPCRs. This strategy yielded multiple extracellular surface-targeting nanobodies that allosterically or competitively modulate AT1R signaling. We then used structure-guided design to successfully convert two allosteric nanobody ligands into competitive antagonists via simple modifications. Our data demonstrate that nanobodies can encode rich and evolvable pharmacological function and have great potential as next-generation GPCR-targeting therapeutics.

## Results

### Epitope-Directed Selection for Nanobody Ligands.

**Fig. 1.**
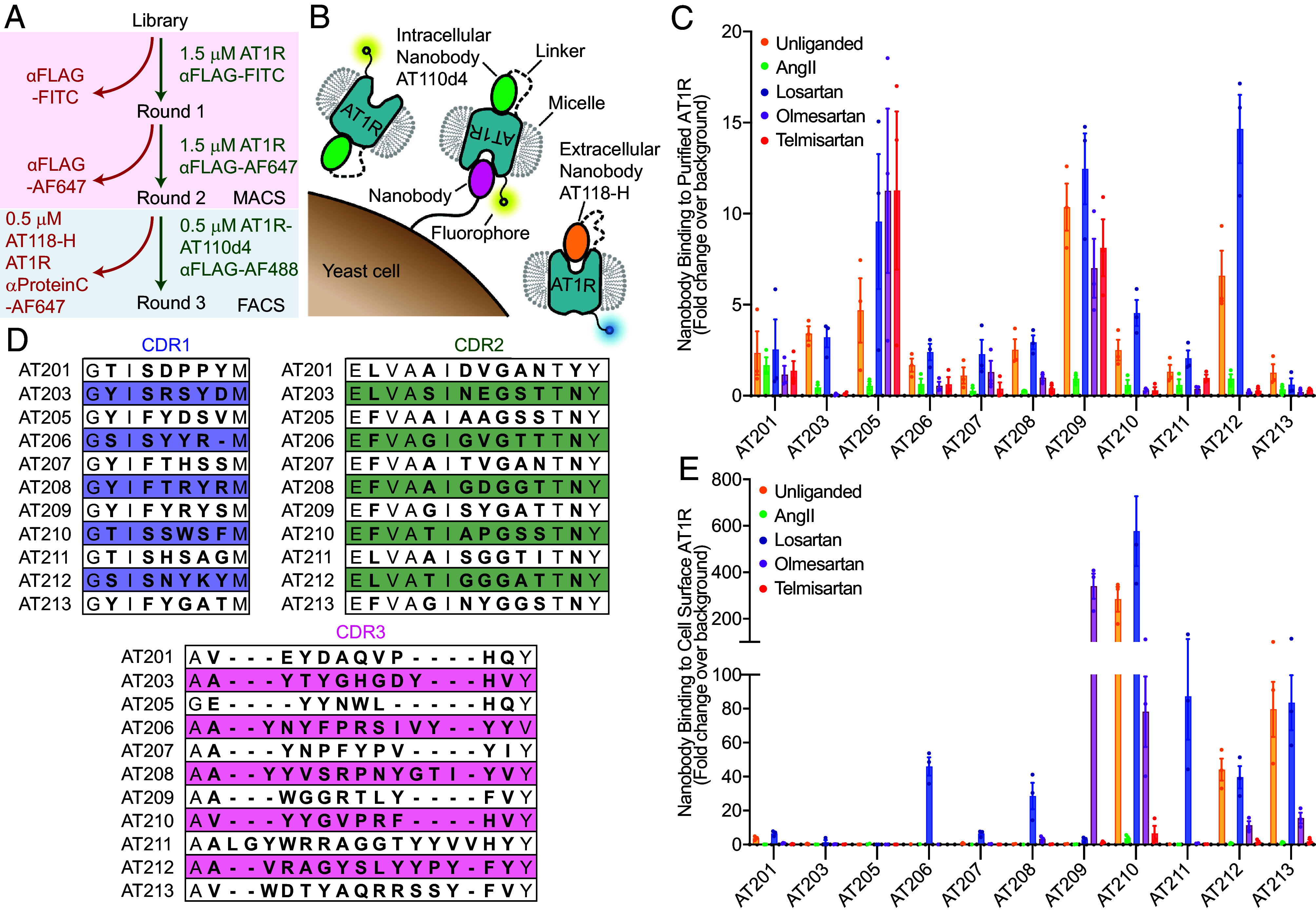
Identification of nanobody ligands for AT1R. (*A*) Flowchart of the selection process. Extracellular-binding nanobodies were enriched from a synthetic nanobody library through two rounds of MACS. In the third round of selection, nanobodies that preferentially bound the extracellular surface of AT1R were sorted via FACS. (*B*) Cartoon of the FACS selection step. Only the extracellular surface of AT1R is accessible for yeast-displayed nanobody binding to the FLAG-AT1R-AT110d4 fusion protein. Yeast-displayed nanobodies binding to the intracellular surface or nanobody framework bind to the AT118-H-AT1R-Protein C fusion protein. Both fusion proteins were labeled with different fluorophore dye-conjugated antibodies for fluorescence-activated cell sorting. The yeast population that preferentially bound to FLAG-AT1R-AT110d4 was collected. (*C*) Binding of single yeast-displayed nanobody clones to 100 nM unliganded, AngII, and antagonist-bound AT1R in detergent. All nanobodies bound to unliganded AT1R and competed for binding with AngII. Variable binding was observed in the presence of AT1R antagonists. Data are presented as mean ± SEM from three experiments. (*D*) CDR sequences of identified nanobodies. Bolded residues were diversified positions in the nanobody library (20). (*E*) Binding of 250 nM purified nanobodies to unliganded, AngII, and antagonist-bound AT1R in intact cell membranes. Nine of 11 nanobodies bound to the extracellular surface of AT1R. Many nanobodies display preferential binding for the antagonist-bound receptor. Data are presented as mean ± SEM from three experiments.

To identify nanobody ligands for AT1R, we enriched a yeast-displayed library of synthetic nanobodies for binders to purified human AT1R using magnetic cell sorting (MACS) (Fig. 1*A*) (20). We then implemented a two-color fluorescence-activated cell sorting (FACS)-based selection with two distinct AT1R fusion proteins to identify nanobody ligands that bind to AT1R’s extracellular surface (Fig. 1*B*). To enrich for nanobody ligands, we positively selected for yeast clones that bound to FLAG-tagged AT1R fused to the intracellular binding nanobody AT110d4 and negatively selected for Protein C-tagged AT1R fused to the extracellular binding nanobody AT118-H (*SI Appendix*, Table S1) (8, 15, 25, 26). We reasoned that nanobodies binding the extracellular face of AT1R would preferentially bind to the FLAG-AT1R-AT110d4 protein, whereas nanobodies binding the intracellular face of AT1R would preferentially bind to the AT118-H-AT1R-Protein C fusion protein. We expected that anti-nanobody nanobodies and nonspecific binders would bind equally to both AT1R fusion proteins. The fusion proteins were labeled with fluorophore-conjugated αFLAG or αProtein C antibodies and 3.7 × 10^4^ cells binding to FLAG-AT1R-AT110d4 were collected in a fluorescence-activated cell sorting (FACS) sort (*SI Appendix*, Fig. S1). The resulting population from the three selection rounds preferentially bound to the AT1R-AT110d4 fusion protein and competed for binding with AT1R’s endogenous ligand angiotensin II (AngII) (*SI Appendix*, Figs. S2 and S3). The ability to compete with an orthosteric ligand suggests that the population contains pharmacologically active extracellular binders (*SI Appendix*, Fig. S2).

We plated out our fluorescence-activated cell-sorted library to obtain single yeast colonies that express a single nanobody and randomly selected 96 colonies for analysis. We then screened single colonies for binding to our positive selection target AT1R-AT110d4 and selected the 43 strongest binding clones for further analysis (*SI Appendix*, Fig. S4). We ensured that these nanobodies retained binding to the WT receptor and not our secondary antibody detection reagents. To eliminate intracellular binders, we counterselected against AT118-H-AT1R. We then screened for nanobodies that interact with AT1R’s orthosteric pocket and compete for binding with AngII (Fig. 1*C* and *SI Appendix*, Fig. S4). About 50% of the clones showed competitive binding with AngII. We selected 14 of these nanobodies for sequencing and obtained 11 nanobody ligands with unique sequences for additional characterization (Fig. 1*D* and *SI Appendix*, Table S2).

### Nanobody Binding Profiles Differ for Detergent-Purified and Membrane-Bound AT1R.

To assess the pharmacological properties of our 11 nanobody ligands, we characterized nanobody binding in the presence of a panel of small-molecule AT1R antagonists that are clinically used for the treatment of high blood pressure. Unlike the peptide agonist AngII, the small-molecule antagonists bind deep within the receptor’s orthosteric pocket and lack interactions with AT1R’s ECLs. We tested for binding both with detergent-purified receptor, which was used in the yeast selection process, and AT1R, the target for nanobody ligands, on intact cells (Fig. 1 *C* and *E* and *SI Appendix*, Fig. S1). We prebound AT1R to saturating concentrations of three AT1R antagonists: losartan, a relatively small modest-affinity antagonist; olmesartan, a high-affinity antagonist which shares a similar chemical structure to losartan and is commercially available as a radioligand probe for AT1R; and telmisartan, a larger high-affinity AT1R antagonist that is predicted to extend upward into the orthosteric binding site where it would likely compete for binding with nanobodies (*SI Appendix*, Fig. S5) (27). We assessed nanobody binding to AT1R by flow cytometry.

We observed distinct binding patterns for the nanobody–receptor pairs in the detergent-purified and on-cell formats, suggesting that the membrane composition or assay setup influences binding properties. All eleven of our nanobodies bound to detergent-purified losartan-bound AT1R at levels similar or higher to unliganded receptor, while only some recognized olmesartan- and telmisartan-bound AT1R (Fig. 1*C*). In the on-cell format 9 of our 11 nanobodies bound to AT1R with at least fivefold signal over background in one or more conditions, confirming that our epitope-directed selection successfully enriched for nanobody ligands (Fig. 1*E*). However, only 3 of our nanobodies bound to unliganded receptor, with the other six recognizing antagonist-occupied AT1R (Fig. 1 *C* and *E*). We observed that some nanobodies bound relatively stronger to on-cell AT1R over detergent-purified AT1R. We attribute this to steric hindrance between the yeast-displayed nanobody and detergent-purified AT1R, which is absent in the on-cell format conducted with soluble nanobody.

The majority of our nanobodies displayed selective binding to losartan-bound AT1R in membranes. Losartan was included during receptor expression and initial purification steps to enhance receptor expression and stability. Purified AT1R was extensively washed to remove the losartan, but trace amounts remaining in the final purified sample may have encoded for recognition of losartan-bound AT1R during the selection process. Intriguingly, AT209 binds exclusively to olmesartan-occupied AT1R in membranes. This result is serendipitous, as olmesartan was not included during receptor purification or any step of the nanobody selection. This selectivity for a particular orthosteric ligand, known as “probe dependence,” is commonly observed among conventional small-molecule allosteric modulators for GPCRs, and has only recently been described for nanobodies (15, 24, 28).

Overall, our selection campaign with detergent-purified AT1R yielded nanobody ligands with preserved binding on cells. However, many nanobodies required the presence of small-molecule antagonists to bind AT1R, suggesting that AT1R samples different distributions of conformational states in detergent and on cells (29). Several nanobodies displayed probe-dependent behavior, binding AT1R in the presence of some, but not all, antagonists. To further explore the mechanistic requirements for nanobody–AT1R binding, we chose two nanobodies, AT206 and AT209, which display distinct binding profiles, for additional characterization.

### AT209 Allosterically Enhances Olmesartan Binding.

**Fig. 2.**
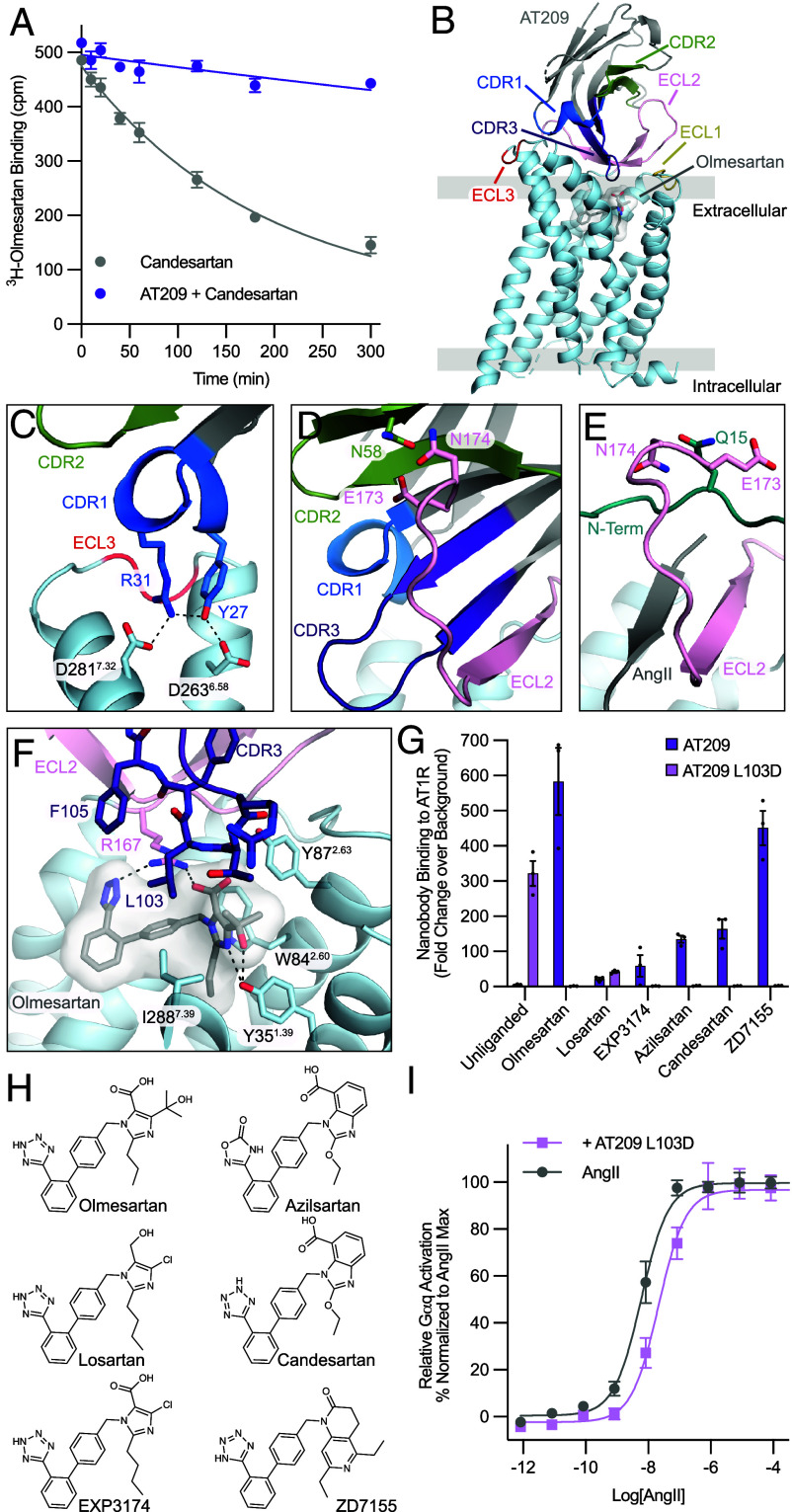
Characterization and engineering of AT209. (*A*) AT209 effects on the dissociation of [^3^H]-olmesartan from AT1R in cell membranes. Data are presented as mean ± SEM from three experiments. AT209 slows the off-rate of olmesartan, indicating that AT209 allosterically potentiates olmesartan binding to AT1R. (*B*) Cryo-EM structure of AT209 in complex with olmesartan-bound AT1R. AT209’s CDRs interact with AT1R’s ECLs. Image is colored by component AT209 (gray), CDR1 (dark blue), CDR2 (light green), CDR3 (purple), AT1R (light blue), ECL1 (yellow), ECL2 (light pink), and ECL3 (red). (*C*) AT209 CDR1 (blue) interacts with acidic residues on TMs 6 and 7. Dashed lines represent hydrogen bonds. (*D*) AT209 CDR3 (purple) forms a continuous β-sheet with AT1R’s ECL2 (light pink). This interaction is stabilized by CDR2 (light green). (*E*) In active-state AT1R structures, AngII (gray) forms a continuous β-sheet with AT1R’s ECL2 (light pink), which is supported by the receptors N terminus (turquoise). (*F*) AT209 CDR3 (purple) sits within AT1R’s olmesartan-bound (gray sticks) orthosteric pocket. L103^CDR3^ sits within a hydrophobic pocket. (*G*) Binding of 250 nM AT209 variants to antagonist-bound AT1R in intact cells. (*H*) Chemical structures of AT1R antagonists. (*I*) AT209 L103D suppress AngII-mediated Gαq signaling, as measured through the NFAT transcriptional reporter. Data are presented as mean ± SEM from three experiments.

In our initial experiments with on-cell AT1R, nanobody AT209 did not bind unliganded, AngII-, losartan-, or telmisartan-bound receptor, but bound to olmesartan-bound AT1R with 100 nM affinity (*SI Appendix*, Table S3). We hypothesized that AT209 may act as a probe-dependent allosteric modulator and potentiate olmesartan’s inhibitory effects (28). We assessed AT209’s ability to enhance olmesartan inhibition of AngII-dependent inositol triphosphate (IP3) production but did not see any clear effect likely due to olmesartan’s already high affinity for AT1R (*SI Appendix*, Fig. S5). A characteristic trait of allosteric modulators is their ability to alter the association and dissociation kinetics of orthosteric ligands (30). Therefore, we determined the dissociation rate of [^3^H]-olmesartan in the presence of candesartan, an insurmountable AT1R antagonist, and AT209 (31). AT209 substantially slowed olmesartan’s dissociation rate, increasing the half-life by 10-fold (Fig. 2*A*), confirming its ability to allosterically enhance the activity of olmesartan.

### AT209 Occludes AT1R’s Orthosteric Pocket.

To interrogate how AT209 acts as an allosteric modulator, we determined a cryo-electron microscopy (cryo-EM) structure of the olmesartan-bound nanobody–receptor complex at 3.0 Å global resolution (Fig. 2*B* and *SI Appendix*, Figs. S6 and S7). As the complex is relatively small for cryo-EM, we enhanced the mass of our sample by fusing thermostabilized apocytochrome b562RIL (BRIL) to the third intracellular loop of AT1R. We then added the BAG2 anti-BRIL Fab and anti-Fab nanobody to further increase the mass and aid in particle alignment (*SI Appendix*, Fig. S6) (15, 32). Density was well resolved for both AT1R and AT209 with resolutions of 2.7 to 3.3 Å at the nanobody receptor interfaces after local refinement (*SI Appendix*, Fig. S7). Overall, the AT209–AT1R–olmesartan complex is in an inactive state, with the receptor core and intracellular face largely resembling the olmesartan-bound crystal structure (r.m.s.d. 0.5 Å for 136 Cα atoms) (33).

Consistent with its ability to slow olmesartan dissociation, AT209 occludes AT1R’s orthosteric pocket trapping the small-molecule antagonist within the receptor’s core (Fig. 2*B*). AT209’s complementarity determining regions (CDRs) bind against the receptor’s extracellular loops (ECLs), with CDR1 and CDR3 driving the nanobody–receptor interaction. CDR1 binds adjacent to transmembrane helices 5 and 6, tilting the two helices and ECL 3 outward from the receptor core. This interaction is stabilized by hydrogen bonds between Y27^CDR1^ and D263^6.58^ as well as a salt bridge between R31^CDR1^ and D281^7.32^ (Fig. 2*C*). CDR3 forms a continuous β-sheet with AT1R’s ECL2, which adopts a β-hairpin fold (Fig. 2 *B* and *D*). This binding mode is reminiscent of the AT1R’s peptide agonist, which also forms a β-sheet with ECL2 (Fig. 2*E*). The AT209–ECL2 interaction is further supported by N58^CDR2^ of AT209, which is well positioned to interact with E173^ECL2^ and N174^ECL2^ on ECL2’s β-hairpin turn. Again, this interaction shares features with agonist-bound AT1R structures, where the ECL2 position is stabilized by an interaction between N15 in AT1R’s N terminus and E173^ECL2^ and N174^ECL2^ in the β-hairpin loop (Fig. 2*E*). Despite its agonist-like binding mode, AT209 requires additional interactions with olmesartan deep within the orthosteric pocket to effectively bind AT1R.

### Basis for the Probe-Dependent Behavior of AT209.

AT209’s CDR3 threads down into AT1R’s orthosteric pocket with its β-turn positioned just above olmesartan. L103^CDR2^ and F105^CDR2^ form a hydrophobic plug above the small-molecule-binding site. Surprisingly, no polar interactions are made between AT209 and olmesartan to drive AT209’s selectivity for olmesartan-bound AT1R over the chemically similar antagonist losartan (Figs. 1*E* and 2 *F* and *G*). Olmesartan and losartan differ in substituents on their imidazole rings, with olmesartan containing a carboxylic acid and tert-butanol and losartan decorated with alcohol and chloride moieties (Fig. 2*H*). To test whether the carboxylic acid-substituted imidazole is required for AT209 binding, we assayed whether AT209 could recognize AT1R bound to EXP3174, a carboxylic acid containing losartan analog (Fig. 2*H*). AT209 bound weakly to EXP3174-bound AT1R, indicating that the carboxylic acid is not sufficient for nanobody binding (Fig. 2*G*).

To gain insight into the structure–activity relationship required for AT209 allostery, we tested whether AT209 could bind AT1R in the presence of a panel of small-molecule AT1R antagonists. We found that AT209 also bound to ZD1755-ocuppied AT1R at similar levels to the olmesartan-occupied receptor. Additionally, azilsartan and candesartan modestly supported AT209 binding (Fig. 2*G* and *SI Appendix*, Fig. S5). We examined the chemical structures of the various antagonists and concluded that AT209 is compatible with ligands containing bulky hydrophobic substituents on the antagonist’s imidazole ring, such as tert-butanol or benzimidazole-carboxylic acid, or the equally bulky naphthyridin-2-one found in the ZD7155 scaffold (Fig. 2*H*). However, extremely bulky antagonists that are predicted to occupy the upper region of the orthosteric pocket, such as EMD66684 and telmisartan, do not bind simultaneously with AT209 as they would clash with CDR3 (*SI Appendix*, Fig. S5) (27).

The tert-butanol, benzimidazole-carboxylic acid, and naphthyridin- 2-one moieties that support AT209 binding are all positioned within a hydrophobic pocket formed between AT1R transmembrane helices 1, 2, and 7, with each group able to accept a hydrogen bond from Y35^1.39^, the sole hydrogen bond donor in the pocket (Fig. 2*F* and *SI Appendix*, Fig. S5) (27). In our AT209-AT1R structure, AT209 L103^CDR3^ sits at the top of this pocket, forming hydrophobic interactions with I288^7.39^ and olmesartan’s tert-butanol group (Fig. 2*F*). Modeling of ZD7155, azilsartan, and candesartan into the ligand binding pocket suggests that L103^CDR3^ could form analogous hydrophobic interactions with these ligands (*SI Appendix*, Fig. S5).

### A Single Amino Acid Substitution Converts AT209 into a Competitive Antagonist.

The structure–activity relationships between AT209 and AT1R antagonists suggest that hydrophobic interactions centered around L103^CDR3^ are responsible for AT209’s probe dependence and allosteric behavior. We reasoned that the introduction of a charged residue would be incompatible with the bulky hydrophobic groups found on olmesartan, ZD7155, candesartan, and azilsartan. Additionally, we hypothesized that an acidic group could form a favorable interaction with R167^ECL2^, which is typically coordinated by AT1R agonists and antagonists, and drastically alter AT209’s pharmacological behavior. We profiled AT209 L103D^CDR3^ binding to AT1R with our full panel of AT1R antagonists (Fig. 2*G* and *SI Appendix*, Fig. S5). As expected, AT209 L103D^CDR3^ does not bind olmesartan-, ZD7155-, candesartan-, and azilsartan-occupied AT1R. Strikingly, AT209 L103D^CDR3^ binds strongly to unliganded AT1R with a comparable affinity to the parent nanobody for olmesartan-bound receptor (*SI Appendix*, Table S3). We evaluated whether AT209 L103D^CDR3^ has different pharmacological behavior from its parent nanobody AT209 in Gαq signaling and β-arrestin recruitment assays (Fig. 2*I* and *SI Appendix*, Fig. S5). AT209 L103D^CDR3^ is an AT1R antagonist, modestly inhibiting AngII-mediated receptor activation. Thus, with a simple single amino acid substitution, we converted AT209 from a primarily allosteric ligand to a fully competitive inhibitor.

### AT206 Is a Selective Allosteric Modulator for Losartan.

**Fig. 3.**
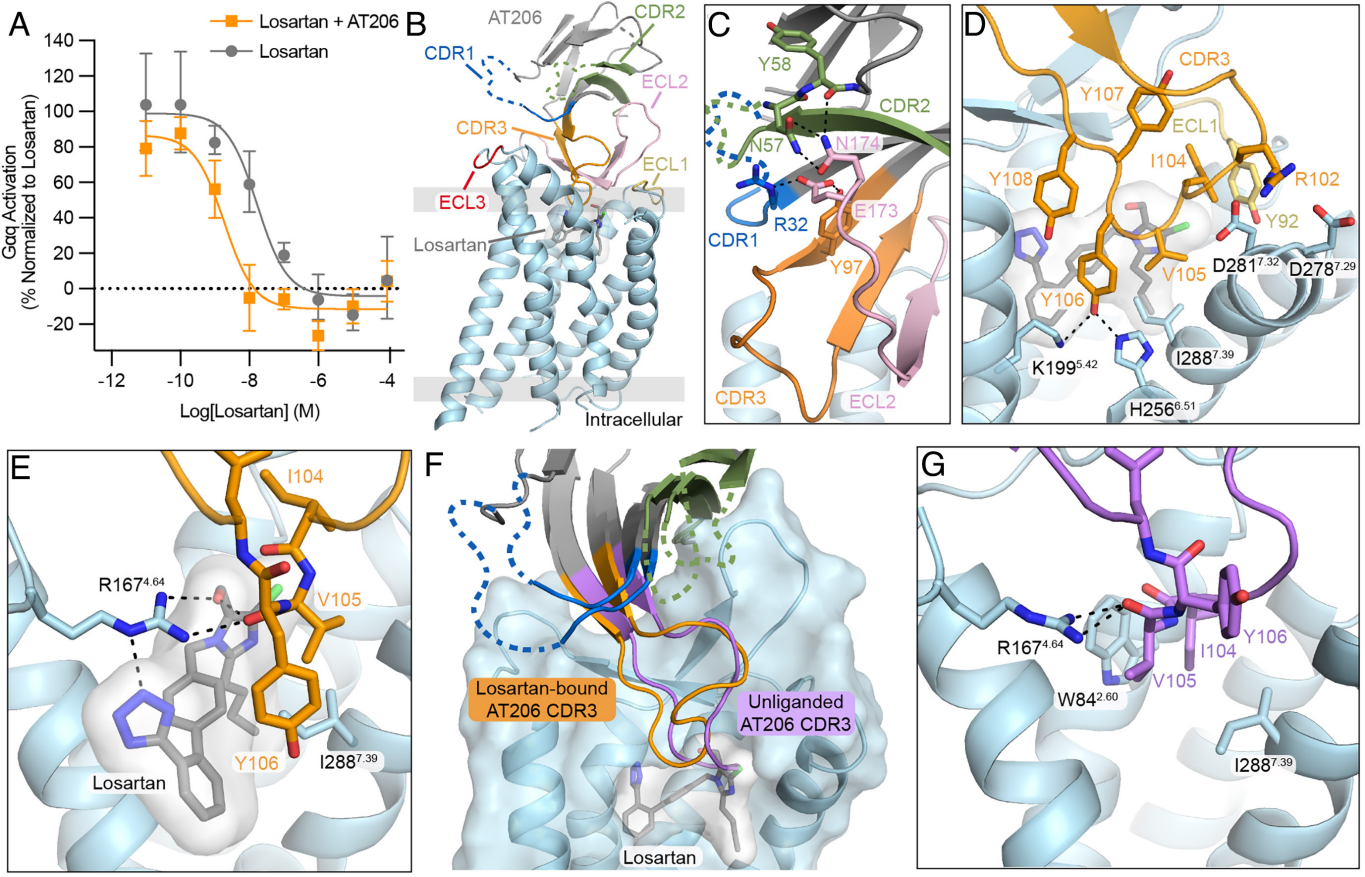
Characterization of AT206. (*A*) AT206 potentiates the inhibitory activity of losartan on AngII-mediated Gαq signaling, as measured through the production of IP3. Data are presented as mean ± SEM from three experiments. (*B*) Cryo-EM structure of AT206 in complex with losartan-bound AT1R. CDR3 enters AT206’s orthosteric pocket, while regions of CDRs 1 and 2 are disordered (dashed lines). Image is colored by component AT206 (gray), CDR1 (dark blue), CDR2 (light green), CDR3 (orange), AT1R (light blue), ECL1 (yellow), ECL2 (light pink), and ECL3 (red). (*C*) AT206 CDR3 (orange) forms a continuous β-strand with ECL2 (light pink). This interaction is supported by CDR2 (light green). Black dashed lines indicate hydrogen bonds. (*D*) CDR3 (orange) interacts with AT1R’s ECL1 (yellow) and TMs 5, 6, and 7. (*E*) The backbone of V105^CDR3^ forms a hydrogen bond network (black dashed lines) with losartan and residues within AT1R’s orthosteric pocket. (*F*) Comparison of AT206 CDR3 in Cryo-EM structures of losartan-bound (orange) and unliganded (purple) AT1R. CDRs 1 and 2 are colored as in (*B*). (*G*) In unliganded AT1R, AT206 CDR3 (purple) occupies losartan’s binding site.

AT209 was the only nanobody in our screen to display probe dependence for olmesartan, but several nanobodies displayed selectivity for losartan-bound AT1R (Fig. 1*E*). Despite their similar binding profiles, AT205, AT207, AT208, and AT211 contain dissimilar CDR sequences (Fig. 1*D*). We were particularly intrigued by AT206 due to its tyrosine-rich CDR3 and selected it for further characterization. We first tested whether AT206 binds compatibly with any other small-molecule AT1R antagonists in our broad ligand panel in mammalian cells. We found that AT206 exclusively binds to losartan-bound AT1R (*SI Appendix*, Fig. S8). EXP3174, a carboxylic acid substituted losartan derivative, does not support AT206 binding, indicating that losartan’s alcohol moiety may interact with the nanobody (Fig. 2*H*). In cell signaling assays, AT206 potentiates losartan inhibition of Gαq signaling, indicating that the nanobody is an allosteric modulator (Fig. 3*A*).

### AT206 CDR3 Interacts Directly with Losartan.

To visualize how AT206 allosterically potentiates losartan, we determined a cryo-EM structure of the losartan-bound AT1R AT206 complex to 3.1 Å global resolution (Fig. 3*B* and *SI Appendix*, Fig. S9). AT206 sits above AT1R’s extracellular face, with the losartan-occupied orthosteric pocket setting the receptor in its inactive state. The nanobody–receptor interaction is largely driven by CDR3, as electron density is weak and, in some regions, nonexistent for CDRs 1 and 2. AT206’s CDR3 binds adjacent to AT1R’s ECL2, forming a continuous β-sheet between the nanobody and receptor’s β-hairpin (Fig. 3 *B* and *C*). This interaction is supported by additional contacts between R32^CDR1^, N57^CDR2^, Y58^CDR2^ on CDRs 1 and 2 and E173^ECL2^ and N174^ECL2^ on the β-turn of ECL2 (Fig. 3*C*). In addition to binding to ECL2, AT206’s CDR3 interacts with other regions of AT1R. R102^CDR3^ is well positioned to interact with D278^7.29^ and D281^7.32^ on TM7 and hydrophobic interactions are formed between I104^CDR3^ and ECL1, and V105^CDR3^ and TM7 (Fig. 3*D*). Hydrophobic residues 104 to 110 of AT206 CDR3 loop over the losartan binding site, trapping the small molecule within AT1R’s orthosteric pocket (Fig. 3*D*). The loop is stabilized through

interactions between AT206 Y106^CDR3^, which binds adjacent to losartan’s biphenyl core, and K199^5.42^ and H256^6.51^ of AT1R (Fig. 3*D*). AT206 also directly interacts with losartan (Fig. 3*E*). The backbone carbonyl of V105^CDR3^ participates in an intricate hydrogen bond network between losartan’s alcohol, tetrazole, and AT1R R167^4.64^ (Fig. 3*E*). This interaction accounts for AT206’s probe dependence for losartan-bound AT1R, as most small-molecule AT1R antagonists contain a carboxylic acid in place of the alcohol, which would be electrostatically incompatible with AT206’s CDR3 (*SI Appendix*, Fig. S8).

### AT206 CDR3 Is Remodeled within the Orthosteric Pocket in Unliganded AT1R.

In our initial selection, AT206 interacted with unliganded detergent-purified AT1R; however, this interaction was not detected in cells, likely due to a reduced affinity for the on-cell receptor (Fig. 1*E*). Given the direct interactions between AT206 and losartan, we suspected that CDR3 would be substantially remodeled within the empty orthosteric pocket and recognize distinct features of AT1R. To visualize this state, we solved an additional structure of the unliganded AT206–AT1R complex to 2.9 Å global resolution (*SI Appendix*, Fig. S10). Despite extensive 3D classification, the overall density for AT206 was poorer than the losartan-bound AT206-AT1R structure, with nearly half of the β-sandwich nanobody framework region disordered; however, CDR3 is well resolved (Fig. 3*F*).

AT206 shares the same overall binding mode for unliganded and losartan-bound AT1R, with CDR3 forming β-stranded interactions with ECL2 and looping down into the orthosteric pocket (Fig. 3*F*). CDR3 binds deeper within the orthosteric pocket in the unliganded structure compared to the losartan-bound AT206–AT1R complex structure. I104^CDR3^ and V105^CDR3^ occupy the losartan binding site and replace some of the interactions that occur between the small molecule and receptor. Similar to the biphenol core of losartan, I104^CDR3^ and V105^CDR3^ form hydrophobic interactions with W84^2.60^ (Fig. 3*G*). In addition, the backbone carbonyl of V105^CDR3^ coordinates R167^4.64^, which forms key interactions with many small-molecule AT1R antagonists (Fig. 3 *E* and *G*) (27, 33).

### CDR Swap Changes AT206’s Pharmacological Properties.

**Fig. 4.**
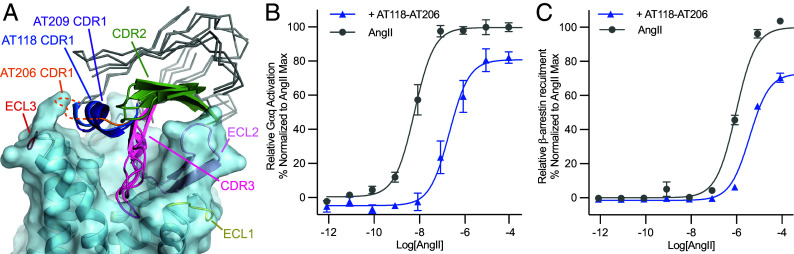
Engineering of AT206. (*A*). Overlay of AT206, AT209, and AT118-bound AT1R. All three CDRs are oriented similarly. Image is colored as follows: CDR1 (AT206, orange; AT209, purple; AT118, dark blue), CDR2 (light green), CDR3 (magenta), AT1R (light blue), ECL1 (yellow), ECL2 (light pink), and ECL3 (red). Unlike the allosteric parent nanobody AT206, the AT118-AT206 chimera suppresses AngII-mediated (*B*) Gαq signaling, as measured through the NFAT transcriptional reporter and (*C*) β-arrestin 2 recruitment, as measured through the TANGO assay. Signaling assay data are presented as mean ± SEM from three experiments.

The overall binding pose of AT206 is shared with AT209 as well as the previously reported AT118 family of nanobodies, which bind to unliganded and antagonist-occupied AT1R and act as both AT1R antagonists and allosteric modulators (8, 15). In all three nanobodies, CDR3 forms a β-sheet with AT1R’s ECL2 and threads into the orthosteric pocket, CDR2 is positioned above ECL2 supporting the nanobody–ECL2 interaction, and CDR1 sits adjacent to ECL3 (Fig. 4*A*). Unlike AT209 and AT118, AT206 CDR1 is weakly or not resolved in our structures, indicating that it is not essential for receptor binding (Fig. 3 *B* and *F*). We compared our AT206–AT1R complex structure with those of the AT118-H AT1R complex and AT118-L in complex with losartan-bound AT1R and predicted that AT118-H CDR1 would be sterically accommodated by AT206 and may enhance the nanobody’s interaction with AT1R.

We replaced AT206 CDR1 with CDR1 from AT118-H and introduced a G26D^CDR1^ stabilizing substitution (34). The resulting AT118-H CDR1-AT206 CDR2 CDR3 chimeric nanobody (AT118-AT206) has a twofold increase in affinity for losartan- bound AT1R (*SI Appendix*, Table S3). Unlike the parent clone, AT118-AT206 also binds to unliganded AT1R with a similar affinity to the losartan-bound receptor (*SI Appendix*, Table S3). We assessed the function of AT118-AT206 in cellular signaling assays and found that the chimeric nanobody is an AT1R antagonist, inhibiting both Gαq signaling and β-arrestin recruitment (Fig. 4 *B* and *C*). Thus, a simple CDR swap between nanobody ligands can effectively enhance affinity and drastically alter pharmacological function.

## Discussion

Small molecules dominate the GPCR therapeutic space, but there has been increasing interest in developing antibody-based GPCR ligands (2). Due to their extended interaction surfaces with antigens, antibodies can recognize nonconserved regions outside of a GPCR’s orthosteric pocket, where most small molecules bind. This fundamentally distinct binding mode allows antibodies to target GPCRs with enhanced specificity over small molecules (7, 15, 16). Furthermore, antibodies are genetically encodable, making them readily amenable to protein engineering strategies to further tune their pharmacological activity and achieve cell and tissue selectivity (7, 15). Still, there are few reported antibodies that directly modulate GPCR signaling.

We developed a strategy to rapidly identify synthetic camelid antibody ligands for AT1R, a prototypical GPCR, from a yeast-displayed nanobody library (Fig. 1 *A* and *B*). Our epitope- directed nanobody selection targets the extracellular face of AT1R, enriching for nanobodies that are more likely to modulate receptor signaling in cells. To enrich for nanobody ligands, we took advantage of existing nanobody tools to block the intracellular and extracellular faces of AT1R (8, 15, 25). This strategy is broadly applicable to other GPCRs where nanobody tools are already available. In cases where nanobodies or antibodies are not available, the intracellular face of the receptor could be blocked with additional tools developed for GPCR structural studies, such as mini-G proteins or fusion proteins (35, 36). We observed distinct binding profiles for yeast-displayed nanobodies and detergent- purified AT1R and soluble nanobodies and on-cell AT1R, suggesting that multiple assays should be considered when prioritizing candidate nanobody clones (Fig. 1 *C* and *E*).

In addition to directly modulating GPCR signaling, we found that nanobody ligands are primed to exert highly specific allosteric effects. Allosteric molecules have been long investigated as a strategy to selectively potentiate or diminish a GPCR’s response to orthosteric ligands (24). Our allosteric nanobody ligands interact with both the ECLs of AT1R and chemical features of the orthosteric ligand. Thus, nanobodies can readily encode specificity for both the receptor and ligand (Figs. 2*F* and 3*E*). We showed that allosteric nanobody ligands can be rapidly identified from pools of nanobody ligands through straightforward binding assays (Fig. 1 *C* and *E*). Alternatively, probe dependence could be directly enriched through additional FACS selection steps.

We found that simple modification of two of our nanobodies, AT206 and AT209, drastically altered their pharmacological function. In the case of AT209, a single amino acid substitution converts the nanobody from an exclusively allosteric to a fully competitive binder, recognizing only unliganded AT1R (Fig. 2*G* and *SI Appendix*, Fig. S5). The AT209 L103D variant is an AT1R antagonist that suppresses AngII-mediated receptor activation (Fig. 2*I*). Structural studies indicated that pharmacology is tuned by CDR3, which enters the orthosteric pocket and binds adjacent to the small-molecule orthosteric ligand (Fig. 2 *B* and *F*). This CDR3-driven selectivity is also seen for other AT1R nanobody ligands (15). We found that additional regions of the nanobody can also influence function. Nanobody AT206 exclusively potentiates the inhibitory effects of losartan (Figs. 1*E* and 3*A* and *SI Appendix*, Fig. S7). Replacing AT206 CDR1 with CDR1 from AT118, another AT1R nanobody antagonist, converted AT206 to a competitive antagonist (Fig. 4 *A*–*C*). AT118 CDR1 binds adjacent to ECL3 and does not enter the orthosteric pocket (15). The CDR1 swap enhances the affinity of AT118-AT206 for unliganded, on-cell AT1R, allowing CDR3 to effectively bind within the unliganded orthosteric pocket (Fig. 3*F* and *SI Appendix*, Table S3).

Overall, we demonstrated that we can rapidly identify nanobody ligands for a GPCR from a synthetic library. These nanobodies can be readily modified through structure-guided protein engineering to alter their pharmacological activity. The two nanobodies characterized in this study, AT206 and AT209, share a binding mode with other GPCR nanobody ligands and allosteric modulators, with an extended CDR3 sitting in or above the orthosteric pocket (7, 11, 12, 14–17). One of these nanobodies, the apelin nanobody antagonist JN-241, was converted to an agonist through a single amino acid insertion to CDR3 (7). Our work further highlights that nanobodies are evolvable pharmacological scaffolds that can modulate GPCR function through both allosteric and competitive mechanisms. Combining nanobody ligands with conventional antibody engineering practices, such as the generation of tissue-specific bispecific molecules, has the potential to modulate GPCR function with a specificity that cannot be readily achieved with small-molecule drugs.

## Materials and Methods

### Nanobody Identification.

Nanobody expression was induced by culturing yeast cells in tryptophan dropout media supplemented with galactose for 36 to 48 h. A library of AT1R-binding nanobodies was prepared through two rounds of magnetic-activated cell sorting (MACS) (25). A total of 1 × 10^10^ cells displaying a synthetic nanobody library were stained with anti-fluorescein isothiocyanate (FITC) microbeads (Miltenyi) and M1-αFLAG antibody conjugated to FITC for 40 min at 4 °C (20). Cells were pelleted and resuspended in selection buffer [20 mM Hepes pH 7.5, 150 mM NaCl, 2.8 mM CaCl2, 0.05% lauryl maltose neopentyl glycol (LMNG), and 0.005% cholesterol hemisuccinate (CHS)]. The yeast were passed over an LD column (Miltenyi) to deplete clones binding to the M1-αFLAG antibody. The remaining yeast were resuspended in selection buffer and incubated with 1 μM FITC conjugated M1-αFLAG antibody and 1.5 μM of FLAG-AT1R for 1 h at 4 °C. Yeast were pelleted and mixed with Anti-FITC microbeads for 15 min and then passed over an LS column (Miltenyi). The column was washed with selection buffer and removed from the magnet. Yeast cells expressing AT1R-binding nanobodies were eluted in selection buffer, pelleted, and amplified in tryptophan dropout media. A total of 4 × 10^8^ yeast cells were subjected to a second round of MACS with AlexaFlour647-labeled M1-αFLAG antibody and anti-AlexaFlour647 microbeads.

To enrich for nanobody ligands, 2 × 10^7^ yeast cells were stained with 0.32 μM AlexaFlour647-conjugated HPC4-αProtein C antibody, 0.5 μM AT118-H-AT1R-Protein C, 0.32 μM AlexaFlour488-conjugated M1-αFLAG antibody, and 0.5 μM FLAG-AT1R-AT110d4 for 1 h at 4 °C. Yeast were washed with selection buffer, and 3.7 × 10^4^ AlexaFlour488-positive cells were collected through FACS on a SONY SH800. The FACS-enriched library was amplified and plated to obtain single clones. Single clones were assessed for binding to FLAG-AT1R-AT110, FLAG-AT1R, and AT118-H-AT1R-Protein C and their ability to compete with the orthosteric ligand AngII.

### Nanobody Expression and Purification.

Nanobodies were cloned into a pET26b vector containing an N-terminal PelB signal sequence for periplasmic expression and a C-terminal V5 epitope followed by a hexahistidine tag. BL21(DE3) *Escherichia coli* cells were transformed with nanobody plasmids and grown in TB supplemented with 4% (v/v) glycerol and 50 μg/mL kanamycin to an OD_600_ of 2 at 37 °C. Cells were cooled to 20 °C for 1 h and induced with 0.2 mM of IPTG. Protein was expressed overnight. Cells were harvested by centrifugation (6,000 × g, 20 min) and cell pellets were stored at −20 °C.

Cell pellets were resuspended in room temperature SET buffer (200 mM Tris pH 8, 500 mM sucrose, 500 μM EDTA) and stirred for 20 min. Resuspended cells were diluted with 2 volumes of cold H_2_O to release the periplasmic contents by osmotic shock and supplemented with 5 mM MgCl_2_ and Benzonase nuclease. The cell mixture was stirred at room temperature for 1 h. Cell debris was pelleted by centrifugation (14,000 × g, 30 min). Then, 100 mM NaCl was added to the supernatant, and the mixture was stirred for 15 min. The supernatant was filtered and passed over Ni-NTA resin. The resin was washed with 10 column volumes of 20 mM Hepes pH 7.5, 500 mM NaCl, 20 mM imidazole followed by 10 column volumes of 20 mM Hepes pH 7.5, 100 mM NaCl, 20 mM imidazole. The nanobodies were eluted in 20 mM Hepes pH 7.5, 100 mM NaCl, and 200 mM imidazole and dialyzed overnight into 20 mM Hepes pH 7.4, 150 mM NaCl, and 10% glycerol. Nanobodies were then concentrated and flash-frozen in liquid nitrogen. Protein purity was assessed by SDS-PAGE.

### Receptor Expression and Purification.

AT1R constructs were cloned into the pcDNA3.1-Zeo-TetO vector containing an N-terminal hemagglutinin signal sequence (*SI Appendix*, Table S4) (37). Wild-type and the AT1R-AT110d4 fusion protein were fused to an N-terminal FLAG tag. A C-terminal protein C tag was included on the AT118-H-AT1R fusion protein for affinity purification. For Cryo-EM, AT206 and AT209 were fused to the N terminus of AT1R followed by a protein C epitope. To enhance sample mass, the cryo-EM constructs contain a thermostabilized apocytochrome b562RIL (BRIL) followed by seven amino acids of helix 6 of the human A2A adenosine receptor and three amino acids from helix 6 of the human frizzled 5 receptor in place of AT1R residues 227 to 233 (15). Expi293F TetR cells grown in Expi293 media were transfected with AT1R plasmids (750 μg/L of culture) with Fectopro (800 μL/L of culture). The culture was supplemented with 3 mM valproic acid and 0.4% glucose 18 h after transfection. Then, 5 μM kifunensine was included in cultures of AT118-H-AT1R-Protein C and the AT206- and AT209-AT1R fusion proteins to limit glycosylation. The culture was supplemented with 5 mM sodium butyrate, and receptor expression was induced with 0.4 μg/mL doxycycline 2 d posttransfection. Then, 1 μM losartan was included in cultures of FLAG-AT1R, FLAG-AT1R-AT110d4 at induction to enhance receptor expression and to the AT206-AT1R fusion protein for the losartan-bound Cryo-EM structure. Cell pellets were harvested 30 h after induction and flash-frozen until purification.

Cell pellets were thawed and resuspended in room-temperature hypotonic lysis buffer (10 mL of 10 mM Tris pH 7.4, 2 mM EDTA, 10 mM MgCl_2_/g cell pellet), with Benzonase nuclease (Sigma Aldrich) and Pierce protease inhibitor tablets (ThermoFisher). Cells were pelleted (50,000 × g, 15 min) and resuspended via Dounce homogenization in (10 mL/g initial cell pellet) cold solubilization buffer (20 mM Hepes pH 7.4, 500 mM NaCl, 0.5% LMNG, 0.05% CHS, 10 mM MgCl2, Benzonase nuclease, and Pierce protease inhibitor tablets). The resuspended cell mixture was stirred for 2 h at 4 °C to solubilize the membranes. For FLAG-AT1R, 1 μM losartan was included during lysis and solubilization to stabilize the receptor. Insoluble material was pelleted by centrifugation (50,000 × g, 30 min). The supernatant was supplemented with 2 mM CaCl2, filtered through a glass fiber filter, and passed over M1-αFLAG or HPC4-αProtein C resin. The resin was washed with 20 column volumes of 20 mM Hepes pH 7.4, 500 mM NaCl, 0.01% LMNG, 0.001% CHS, and 2 mM CaCl_2_ and eluted with 20 mM Hepes pH 7.4, 500 mM NaCl, 0.01% LMNG, 0.001% CHS, 5 mM EDTA with 0.2 mg/mL FLAG (DYKDDDDK), or Protein C peptide (EDQVDPRLIDGK). The receptor was concentrated in a 50 kDa cutoff Amicon-Ultra centrifugal filter and loaded onto a Superdex S200 (10/300) Increase column in 20 mM Hepes pH 7.4, 100 mM NaCl, 0.01% LMNG, and 0.001% CHS.

For the ligand-bound AT206-AT1R and AT209-AT1R cryo-EM structures 1 μM of losartan or olmesartan was included in all purification buffers. The C-terminal tag was removed from protein used for the unliganded AT206-AT1R structure and olmesartan-bound AT209-AT1R structure with 3C protease (1:10, w/w ratio), and the receptor was deglycosylated with endoH (1:10, w/w ratio) prior to size exclusion chromatography.

### Fab Expression and Purification.

The BAG2 (anti-BRIL) Fab heavy chain was cloned into pTarget (Promega), and light chain was cloned into pD2610 (ATUM) (32). Expi293F TetR cells in Expi293 media were transfected with heavy and light chain plasmids (375 μg each/L of culture) with Fectopro (800 μL/L of culture). The culture was supplemented with 3 mM valproic acid and 0.4% glucose 18 to 24 h posttransfection. After 5 to 7 d, the supernatant was collected and applied to CaptureSelect IgG-CH1 resin (ThermoFisher). The resin was washed with 10 column volumes of 20 mM Hepes pH 7.4 and 150 mM NaCl. The Fab was eluted with 100 mM glycine pH 2.5 and neutralized immediately with 1 M Hepes pH 8 (7:3, v/v ratio). The Fab was dialyzed overnight into 20 mM Hepes pH 7.4, 150 mM NaCl, and 10% glycerol, concentrated, and flash-frozen.

### Cryo-EM Sample Preparation.

The nanobody–AT1R fusion proteins were mixed with 1.4-fold molar excess of the BAG2 anti-BRIL Fab and twofold molar excess of an anti-Fab nanobody. The complex was formed on ice for 1 h. Then, 2 mM CaCl_2_ was added to the mixture, and the protein was loaded onto 1 mL of HPC4-αProtein C resin. The resin was washed with 6 column volumes of 20 mM Hepes pH 7.4, 100 mM NaCl, 0.01% LMNG, 0.001% CHS, and 2 mM CaCl_2_. The samples were then washed with 20 column volumes of 20 mM Hepes pH 7.4, 100 mM NaCl, 0.05% glyco-diosgenin (GDN), 0.005% CHS, and 2 mM CaCl_2_ to exchange the detergent. The complex was eluted with 20 mM Hepes pH 7.4, 100 mM NaCl, 0.05% GDN, 0.005% CHS + 5 mM EDTA + 0.2 mg/mL Protein C peptide and concentrated in a 100 kDa cutoff Amicon-Ultra centrifugal filter. The complex was further purified by size exclusion chromatography (Superdex S200 Increase 10/300) in 20 mM Hepes pH 7.4, 100 mM NaCl, 0.05% GDN, and 0.005% CHS. Peak fractions were concentrated to 6 to 9 mg/mL. For small-molecule antagonist-bound complexes, 5 μM of the antagonist was included in all buffers.

Three microliters of the concentrated protein sample was applied to glow-discharged UltraAuFoil 300 mesh grids with 1.2 μm diameter/1.3 μm spacing (TedPella) at 20 °C with 100% humidity. Grids were blotted for 4 to 7 s with a blot force of 15 on a Vitrobot Mark IV Vitrobot (ThermoFisher) and then plunged into liquid-nitrogen-cooled liquid ethane.

### Cryo-EM Structure Determination.

Cryo-EM data were collected in counted mode on a 300 kV Titan Krios G3i microscope (ThermoFisher) equipped with a K3 detector (Gatan) and GIF quantum energy Filter (20 eV) (Gatan) at the Harvard Cryo-Electron Microscopy Center for Structural Biology. SerialEM data collection software v4.05 was used to collect one movie from the center of each hole (38). Nine holes were visited per stage position. Data collection parameters are listed in *SI Appendix*, Table S5.

Data processing was carried out in CryoSparc v3 and v4 (*SI Appendix*, Figs. S7, S9, and S10) (39). Movies underwent patch motion correction, dose-weighting, and patch contrast transfer function (CTF) estimation. Particles were picked with the blob-picker or template-picker functions. One round of 2D classification was used to identify a subset of particles to generate ab initio reconstructions. All particles were then classified through multiple rounds of heterogenous refinement followed by nonuniform refinement with global CTF refinement. Images were then subjected to reference-based motion correction followed by additional rounds of heterogenous refinement and nonuniform refinement. A local mask excluding the anti-Fab nanobody and CH1 and CL domains of the anti-BRIL Fab was applied for local refinement. A model of the complex was built in Coot and refined with Phenix real-space refinement (40, 41). In regions with strong density for the protein backbone, but weak density for side chains, side chains were modeled with the most common rotamer position that did not clash with the model. Local resolution estimates were calculated in Phenix. Figures were prepared in UCSF Chimera and Pymol v9.5.0 (42, 43). All structural biology software was compiled by SBGrid (44).

### Yeast Flow Cytometry Binding Assays.

A total of 1 × 10^6^ yeast cells from a selection round or single clone were washed in selection buffer and stained with 500 nM of receptor and 320 nM of fluorophore-conjugated M1-αFLAG or HPC4-αProtein C antibody (library) or 100 to 150 nM of receptor and 75 to 100 nM of fluorophore-conjugated M1-αFLAG or HPC4-αProtein C antibody (single clone) in 50 μL volume of selection buffer. Yeast were stained with 1:500 (v/v) of 1 mg/mL fluorophore-conjugated αHA antibody to evaluate nanobody expression. Staining reactions were incubated at 4 °C for 30 min and washed with selection buffer. Yeast cells were gated and assessed for nanobody expression and receptor binding on a CytoFLEX flow cytometer using the CytExpert software v2.4.0.28 (*SI Appendix*, Fig. S1). For assays with ligand-occupied AT1R, 1 μM of the ligand was included in the staining reactions and in all washes. Data represent the mean and SE of three independent experiments.

### Flow Cytometry Assays on Mammalian Cells.

To assess nanobody binding to membrane-bound AT1R, Expi293F TetR cells stably expressing FLAG-AT1R under a tetracycline-inducible promoter were induced at 2 × 10^6^ cells/mL with 0.4 μg/mL doxycycline hyclate for 16 to 20 h (34). A total of 1.4 × 10^5^ cells were pelleted (300 × g, 4 min) and washed with flow assay buffer (20 mM Hepes pH 7.4, 150 mM NaCl, + 0.1% BSA). Cells were preincubated with 10 μM of ligand at 4 °C for 30 min in a 50 μL volume. Nanobody was added, bringing the total assay volume to 100 μL. Cells were incubated with ligand and nanobody for 1 h and then washed twice with flow assay buffer containing 5 μM of the appropriate ligand. Assay mixtures were then stained with 100 nM M1-αFLAG-AlexaFlour488 antibody and 1:500 (v/v) αV5-AlexaFluor647 (Thermo Fisher) in flow assay buffer supplemented with 2 mM CaCl_2_. Cells were washed once, resuspended with flow assay buffer with 2 mM CaCl_2_ and 5 μM ligand (100 μL volume), and incubated for 20 min at 4 °C. Cells were washed once and resuspended in flow assay buffer supplemented with 2 mM CaCl2 with 5 μM ligand (50 μL volume). Nanobody binding was assessed on a CytoFLEX flow cytometer (Beckman Coulter). Single cells were gated for receptor expression using the CytExpert software v2.4.0.28 (*SI Appendix*, Fig. S1), and data were analyzed using GraphPad Prism v9.5.1. For single-point assays, cells were stained with 250 nM or 500 nM of nanobody. Data represent the mean and SE of three independent experiments.

### Radioligand Competition Assays.

First, 20 to 50 ng of purified FLAG-AT1R or FLAG-AT1R-AT110d4 was incubated with 5 nM [^3^H]-olmesartan and varying amounts of AngII in 20 mM Hepes pH 7.4, 100 mM NaCl, 0.01% LMNG, 0.001% CHS, and 0.1% BSA for 90 min at room temperature in 200 μL total volume. Then, reactions were harvested on GF/B filters soaked in water and washed three times with a 96-well Brandel harvester. Data were fit using a one-site competition binding model in GraphPad Prism. Radioligand affinity (K_d_) was determined for each construct through saturation binding with varying amounts of [^3^H]-olmesartan to calculate inhibitory constant (K_i_) values from competition binding data. Saturation binding data were fit with the one-site saturation-binding model. Nonspecific binding of [^3^H]-olmesartan was measured in the presence of 10 μM candesartan. Data represent the mean and SE of three independent experiments.

### Radioligand Dissociation Assays.

To measure [^3^H]-olmesartan dissociation from AT1R, membranes were prepared from Expi293F TetR cells stably expressing FLAG-AT1R under a tetracycline-inducible promoter. Measurements were carried out at room temperature in 50 mM Tris pH 7.4, 12.5 mM MgCl_2_, 150 mM NaCl, and 0.2% BSA in 200 μL total volume. Membranes were prelabeled with 2 nM [^3^H]-olmesartan for 45 min. Then, 5 μM of purified AT209 was added, and the mixture was incubated for an additional 45 min. Dissociation of the radioligand was initiated through the addition of candesartan (10 μM final concentration). Reactions were harvested on GF/B filters soaked in water with a 96-well Brandel harvester and washed with cold water three times. Dissociation data were fit in GraphPad Prism with a one-phase exponential decay model. The baseline was constrained to the experimentally determined nonspecific binding of [^3^H]-olmesartan in the presence of 10 μM candesartan.

### Cellular Signaling Assays.

#### Gαq inhibition.

Expi293F TetR cells stably expressing tetracycline-inducible wild-type human FLAG-AT1R were induced with 0.4 μg/mL doxycycline hyclate at 2 × 10^6^ cells/mL for 18 to 20 h. A total of 2 × 10^4^ cells were plated into a low-volume 96-well plate in Expi293 media and pretreated with varying concentrations of olmesartan or losartan for 30 min. Then 5 μM of nanobody ligand was added and the cells were incubated for an additional 30 min. Cells were then stimulated with 10 nM of AngII for 1 h. Levels of IP1 were measured with the IP-One Gq kit (CisBio) and read out on a SpectraMax M5e plate reader.

#### Gαq activation.

HEK293T cells were stably transfected with a plasmid encoding wild-type human FLAG-AT1R fused to an N-terminal hemagglutinin signal sequence (pTarget with the neomycin resistance gene replaced with the puromycin resistance gene). After puromycin selection, single cells were obtained by limited dilution, and a monoclonal line with high levels of FLAG-AT1R expression was selected for further experiments. The FLAG-AT1R HEK293T line was grown in DMEM + 10% FBS and transfected with 3.5 μg of a NFAT luciferase reporter plasmid [gift from Toren Finkel (National Institutes of Health, Bethesda, MD), Addgene plasmid #10959 (45)] with FuGENE (3 μL/μg DNA) in a 10 cm plate. Eighteen hours after transfection, cells were lifted with trypsin, and 4-5 × 10^4^ cells were plated in poly-D-lysine-coated 96-well clear bottom plates in serum- and phenol red-free DMEM. After 6 h, cells were incubated with 5 μM nanobody ligands for 1 h. Cells were then stimulated with varying amounts of AngII overnight. Luciferase expression was read out with the Bright-Glo Luciferase Assay System (Promega) on a SpectraMax M5e plate reader (Molecular Devices).

#### β-arrestin2 recruitment.

A TANGO assay was used to monitor the recruitment of β-arrestin2 to AT1R (46). Wild-type human AT1R was fused to an N-terminal hemagglutinin signal sequence followed by a FLAG tag and C-terminal TEV protease site followed by the tTA transcriptional activator and cloned into the pTarget vector (Promega). HTLA cells stably expressing the tTA-dependent luciferase reporter and the β-arrestin2-TEV protease fusion protein were grown in DMEM + 10% FBS and transfected with 10 μg of the FLAG-AT1R-tTA plasmid with FuGENE [3 μL/μg DNA (46)] in a 10 cm plate. Cells were lifted with trypsin 18 h after transfection and 4-5 × 10^4^ cells were plated in poly-D-lysine coated 96-well clear bottom plates in serum- and phenol red-free DMEM. After 6 h, cells were incubated with nanobody ligands for 1 h. Cells were then stimulated with varying amounts of AngII overnight. Luciferase expression was read out with the Bright-Glo Luciferase Assay System (Promega) on a SpectraMax M5e plate reader (Molecular Devices).

## Supplementary Material

Appendix 01 (PDF)

## Data Availability

The cryo-EM model and maps were deposited under the following accession numbers: AT209 in complex with olmesartan-bound AT1R PDB: 9EAH (47), EMDB: EMD-47831 (48); AT206 in complex with losartan-bound AT1R PDB: 9EAI (49), EMDB: EMD-47832 (50); and AT206-AAT1R complex PDB: 9EAJ (51), EMDB: EMD-47833 (52). All other data are included in the manuscript and/or *SI Appendix*.
