# Supplementary material for: Epitope-directed selection of GPCR nanobody ligands with evolvable function
Source: Proc Natl Acad Sci U S A. 2025 Mar 11;122(11):e2423931122. doi: 10.1073/pnas.2423931122 (PMC11929449; doi:10.1073/pnas.2423931122)
Supplement: Supplementary file 1 — Appendix 01 (PDF) [file pnas.2423931122.sapp.pdf]

PNAS

Supporting Information for

Epitope-Directed Selection of GPCR Nanobody Ligands with Evolvable Function

Meredith A. Skiba, Clare Canavan, Genevieve R. Nemeth, Jinghan Liu, Ali Kanso, Andrew C. Kruse

*Corresponding Author: Andrew C. Kruse
Email: Andrew_Kruse@hms.harvard.edu

This PDF file includes:

Figures S1 to S10
Tables S1 to S5

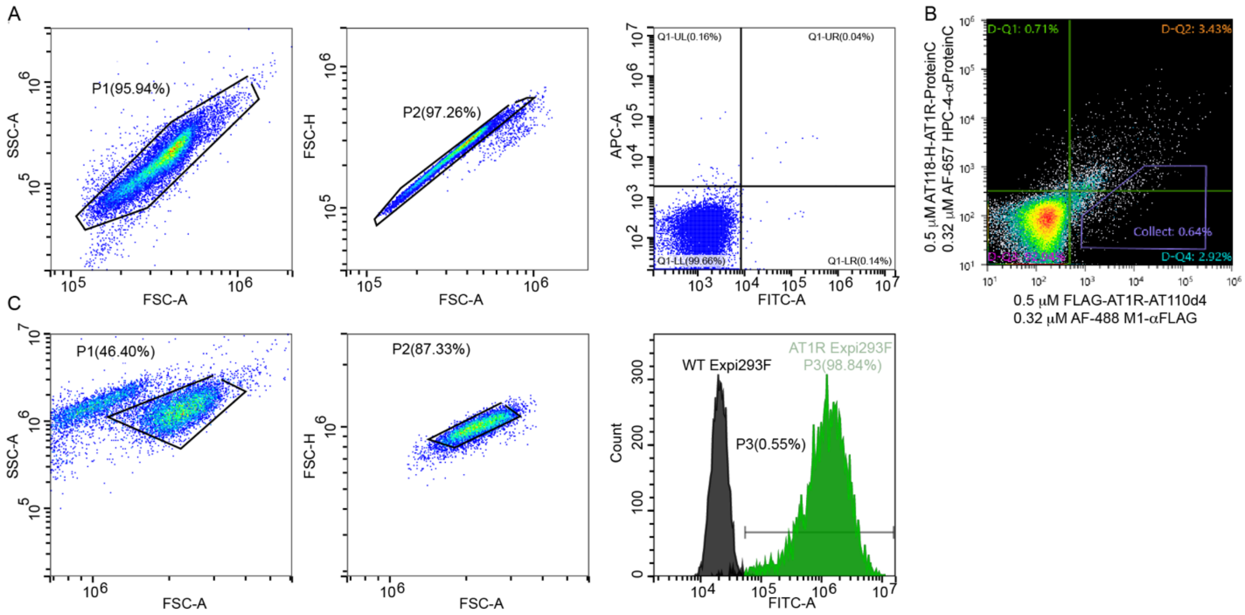

Fig. S1. Representative flow cytometry gating. A) Flow cytometry plots illustrating gating strategy for yeast cells. Data is for uninduced yeast that lack nanobodies displayed on the cell surface. B) Fluorescence-activated cell sorting (FACS) strategy to obtain nanobody ligands. 0.64% of the population was collected. C) Flow cytometry plots illustrating gating strategy for Expi293F cells stably expressing FLAG-AT1R. AT1R expression was monitored with M1-αFLAG-AF488.

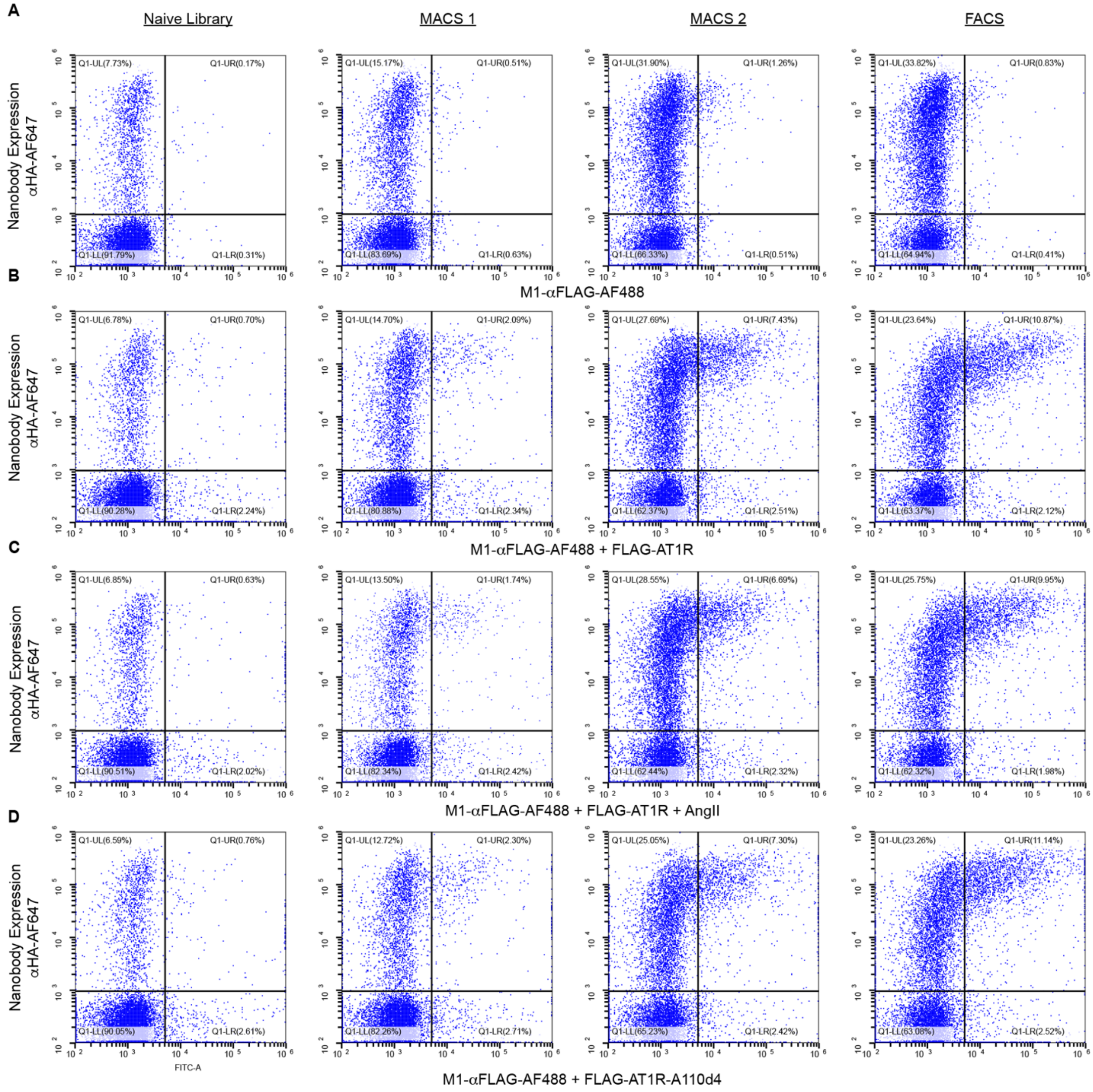

Fig. S2. Analytical staining of nanobody selection rounds. The naïve library, MACS1 population, MACS2 population, and FACS population were stained with αHA-AF647 to monitor for nanobody expression and A) 320 nM M1-αFLAG-AF488 secondary detection reagent, B) 500 nM FLAG-AT1R, C) 500 nM FLAG-AT1R + AngII, D) 500 nM FLAG-AT1R-AT110d4. AT1R nanobody binders are enriched throughout the selection process.

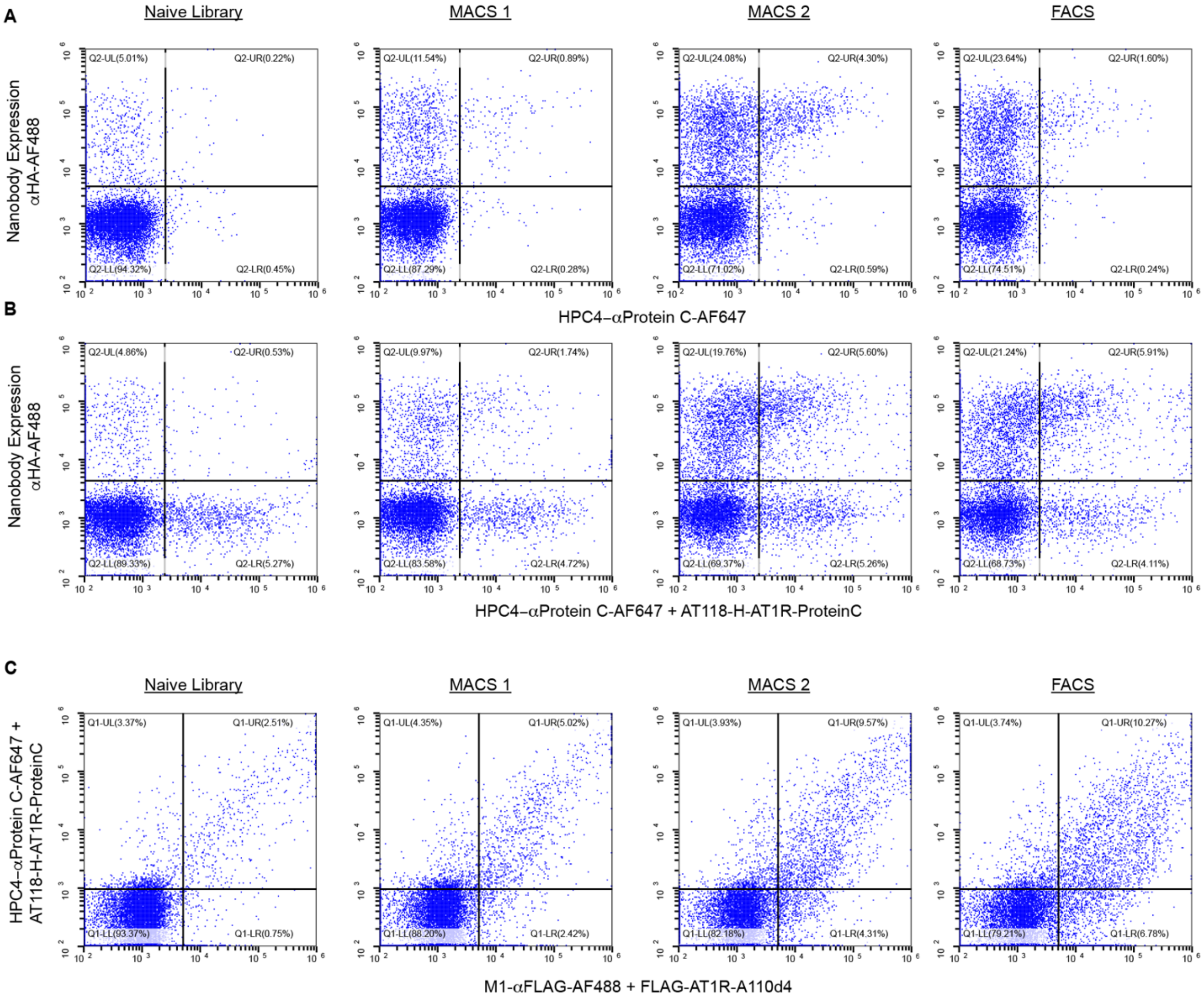

Fig. S3. Analytical staining of nanobody selection rounds. The naïve library, MACS1 population, MACS2 population, and FACS population were stained with A-B) αHA-AF488 to monitor for nanobody expression and A) the HPC4-αProtein C-AF647 detection reagent (320 nM) and B) the negative selection fusion protein AT118-H-AT1R-Protein C (500 nM). HPC4-αProtein C-AF647 and AT118-H-AT1R Protein C display modest levels of non-specific binding to yeast and the nanobody library. C) Dual staining of nanobody selection rounds with the positive selection fusion protein FLAG-AT1R-AT110d4 (500 nM) and the negative selection fusion protein AT118-H-AT1R-Protein C (500 nM). The FACS sorted population is enriched for clones that recognize FLAG-AT1R-AT110d4 (bottom right quadrant).

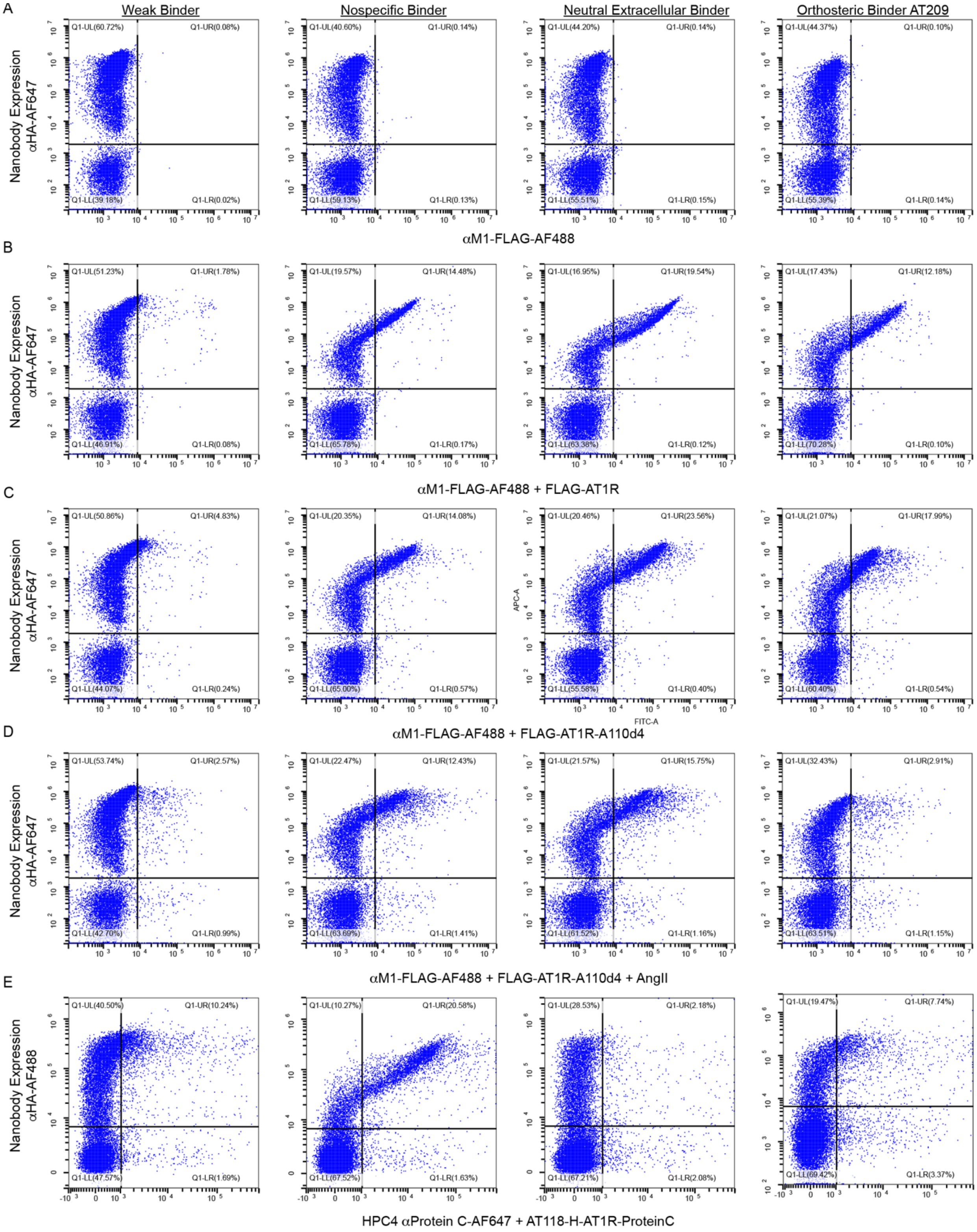

Fig. S4. Representative staining of single nanobody clones. Binding profiles are shown for: weak AT1R binders; non-specific binders that recognize both the extracellular and intracellular face of AT1R; neutral binders recognizing AT1R's extracellular region, but not competing with the orthosteric ligand AngII; and extracellular binders that interact with the orthosteric site. Yeast cells

were stained with αHA-647 to monitor nanobody expression and A) 105 nM M1-αFLAG-AF488 secondary detection reagent, B) 150 nM FLAG-AT1R, C) 100 nM FLAG-AT1R-AT110d4 D) 100 nM FLAG-AT1R-AT110d4 + AngII or E) αHA-488 to monitor nanobody expression and 150 nM AT118-H-AT1R Protein C conjugated to the HPC4-αProtein C-AF647detection reagent (105 nM).

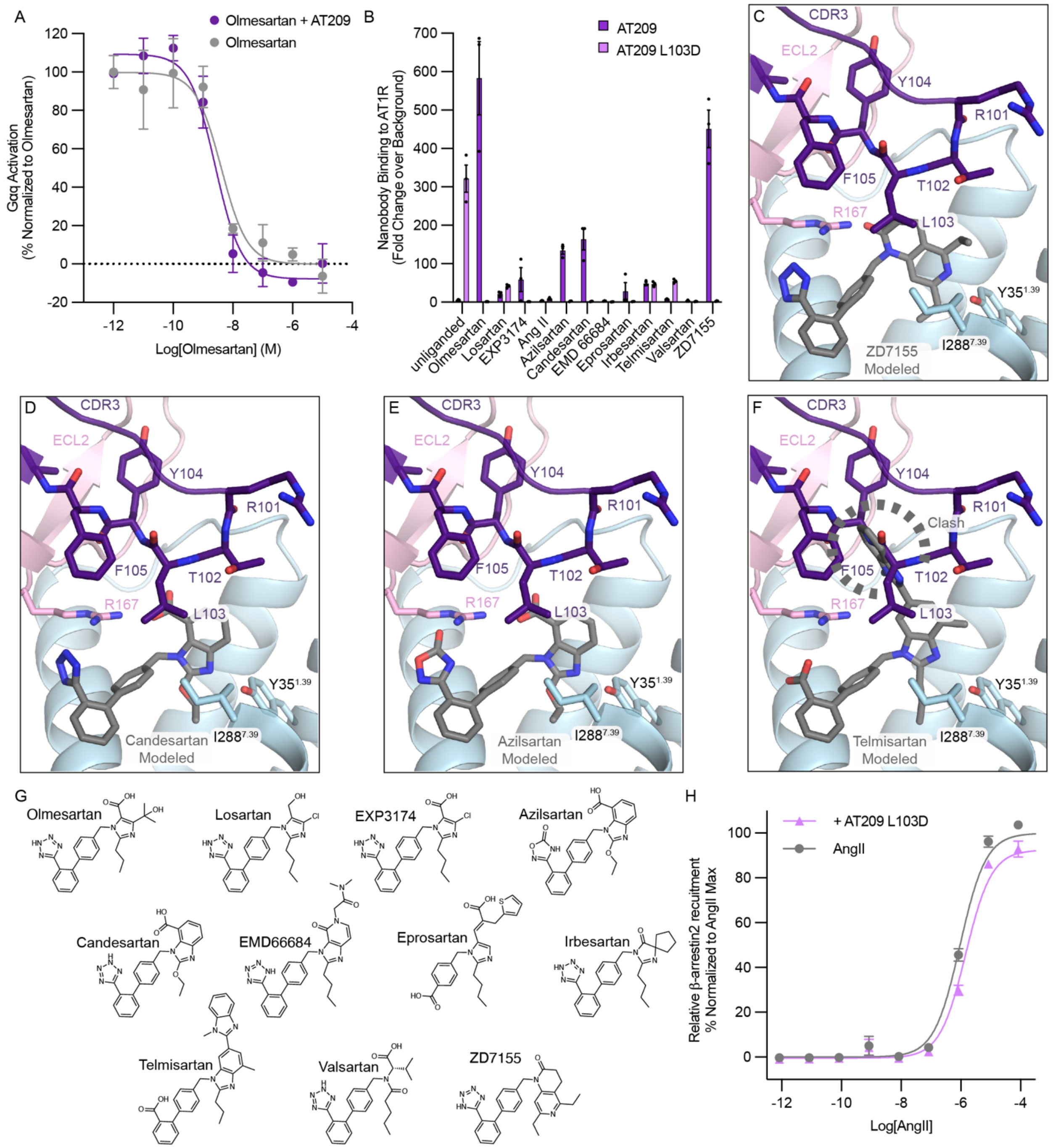

Fig. S5. Expanded characterization of nanobody AT209. A) AT209 fails to potentiate the inhibitory activity of Olmesartan on AngII mediated Gαq signaling, as measured through the production of IP3. B) Binding of 250 nM AT209 variants to ligand-bound AT1R in intact cells. AT209 in complex with AT1R with C) modeled ZD7155, D) modeled candesartan, and E) modeled azilsartan, which support AT209 binding. F) modeled telmisartan clashes with AT209 CDR3. G) chemical structures of AT1R antagonists. H) AT209 L103D weakly suppresses AngII mediated β-arrestin 2 recruitment, as measured through the TANGO assay. Signaling assay data are presented as mean ± s.e.m from three experiments.

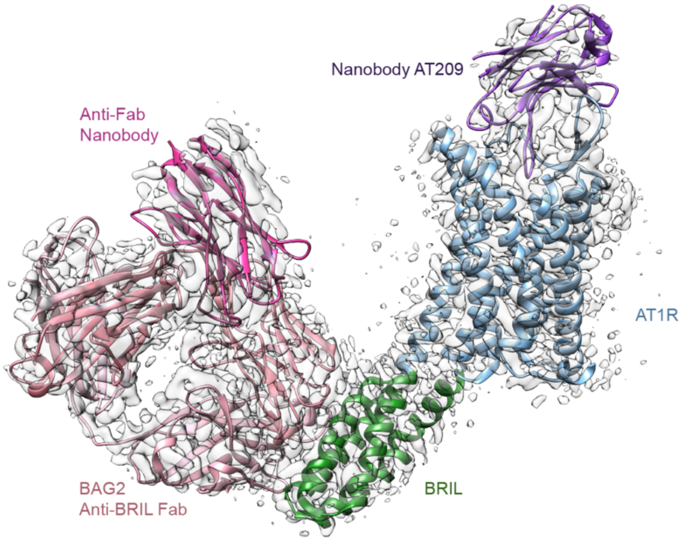

Fig. S6. Construct components for Cryo-EM data collection. To enhance the sample mass for particle alignment, the third extracellular loop of AT1R (blue) was replaced with a rigidly fused BRIL and complexed with the BAG2 anti-BRIL Fab (pink) and an anti-Fab nanobody (magenta). A local mask excluding the anti-Fab nanobody and CH1 and CL domains of the Anti-BRIL Fab was applied for local refinement to generate the final maps for model building. The global density map for the AT209-AT1R complex is shown in white.

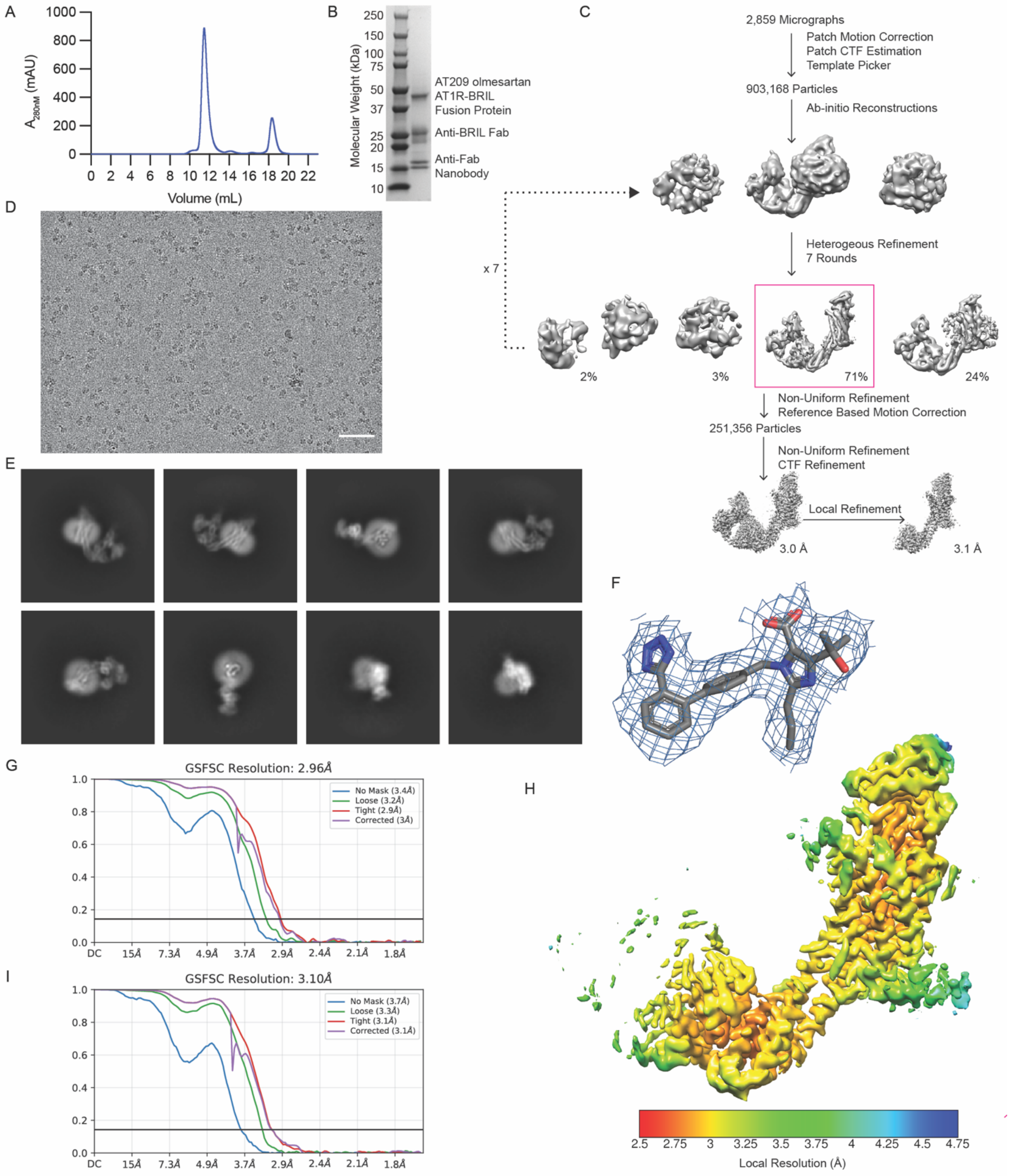

Fig. S7. AT209-AT1R olmesartan data processing. A) Size exclusion trace, B) SDS-PAGE gel under reducing conditions, C) cryo-EM data processing scheme, D) representative micrograph (scale bar = 50 nm), and E) representative 2D class averages of AT209-AT1R-BRIL, anti-BRIL Fab, anti-Fab nanobody complex. F) experimental density for olmesartan. G) Fourier shell correlation (FSC) used to determine global map resolution. H) Local resolution estimate of final map. I) FSC used to determine locally refined map resolution.

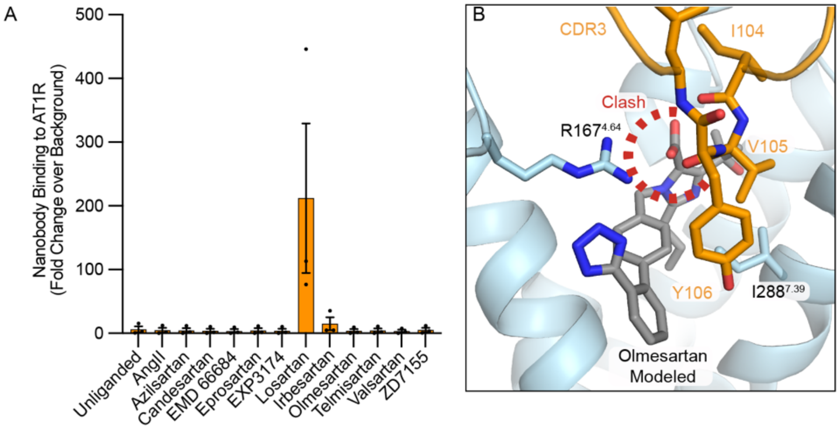

Fig. S8. Expanded characterization of nanobody AT206. A) Binding of 500 nM AT206 to ligand-bound AT1R in intact cells. Error represents the s.e.m. determined from three independent experiments B) The carboxylic acid found on many AT1R antagonists like olmesartan electrostatically clashes with the backbone of AT206 CDR3.

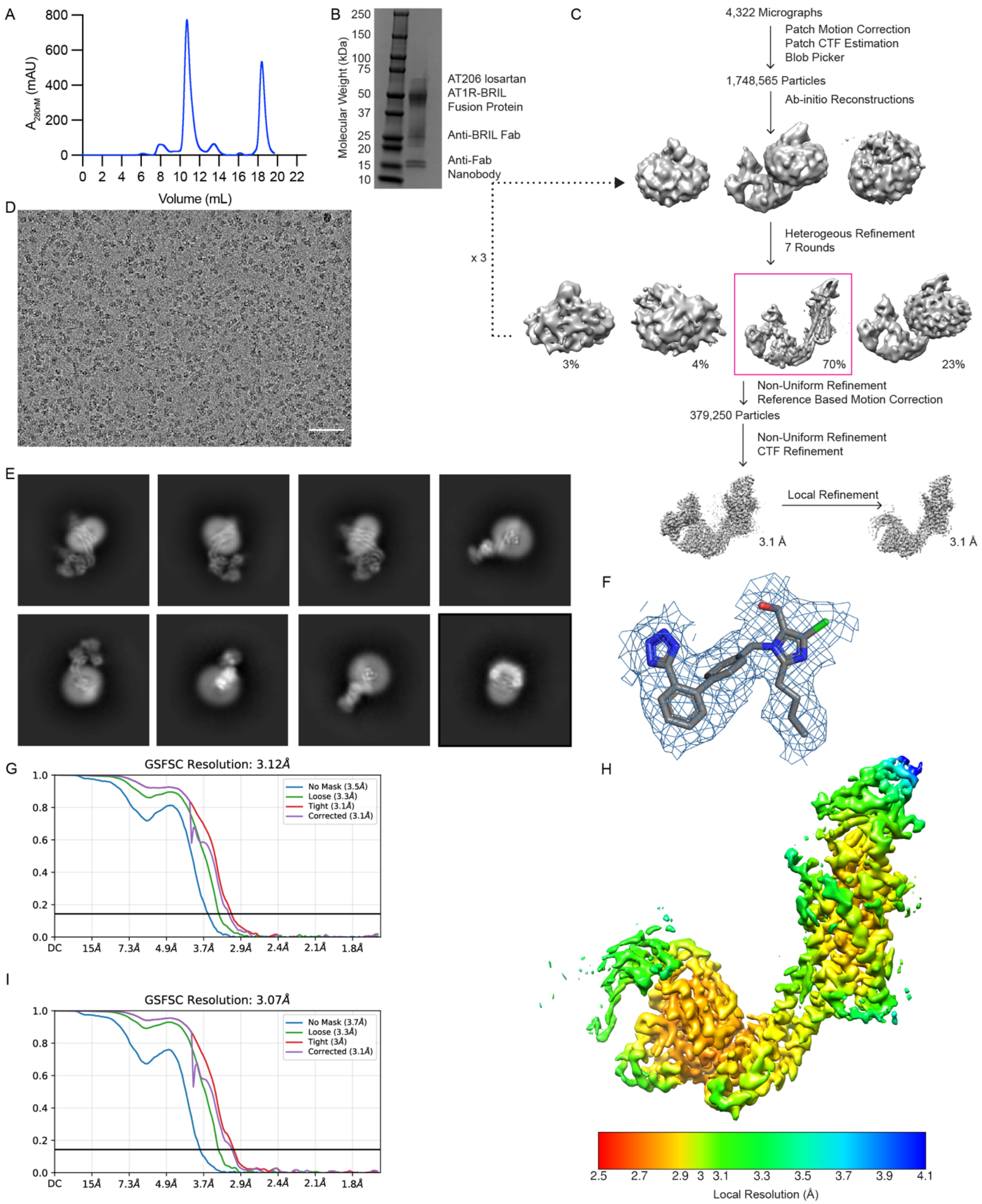

Fig. S9. AT206-AT1R losartan data processing. A) Size exclusion trace, B) SDS-PAGE gel under reducing conditions, C) cryo-EM data processing scheme, D) representative micrograph (scale bar = 50 nm), and E) representative 2D class averages of AT206-AT1R-BRIL, anti-BRIL Fab, anti-Fab nanobody complex. F) experimental density for losartan. G) Fourier shell correlation (FSC) used to determine global map resolution. H) Local resolution estimate of final map. I) FSC used to determine locally refined map resolution.

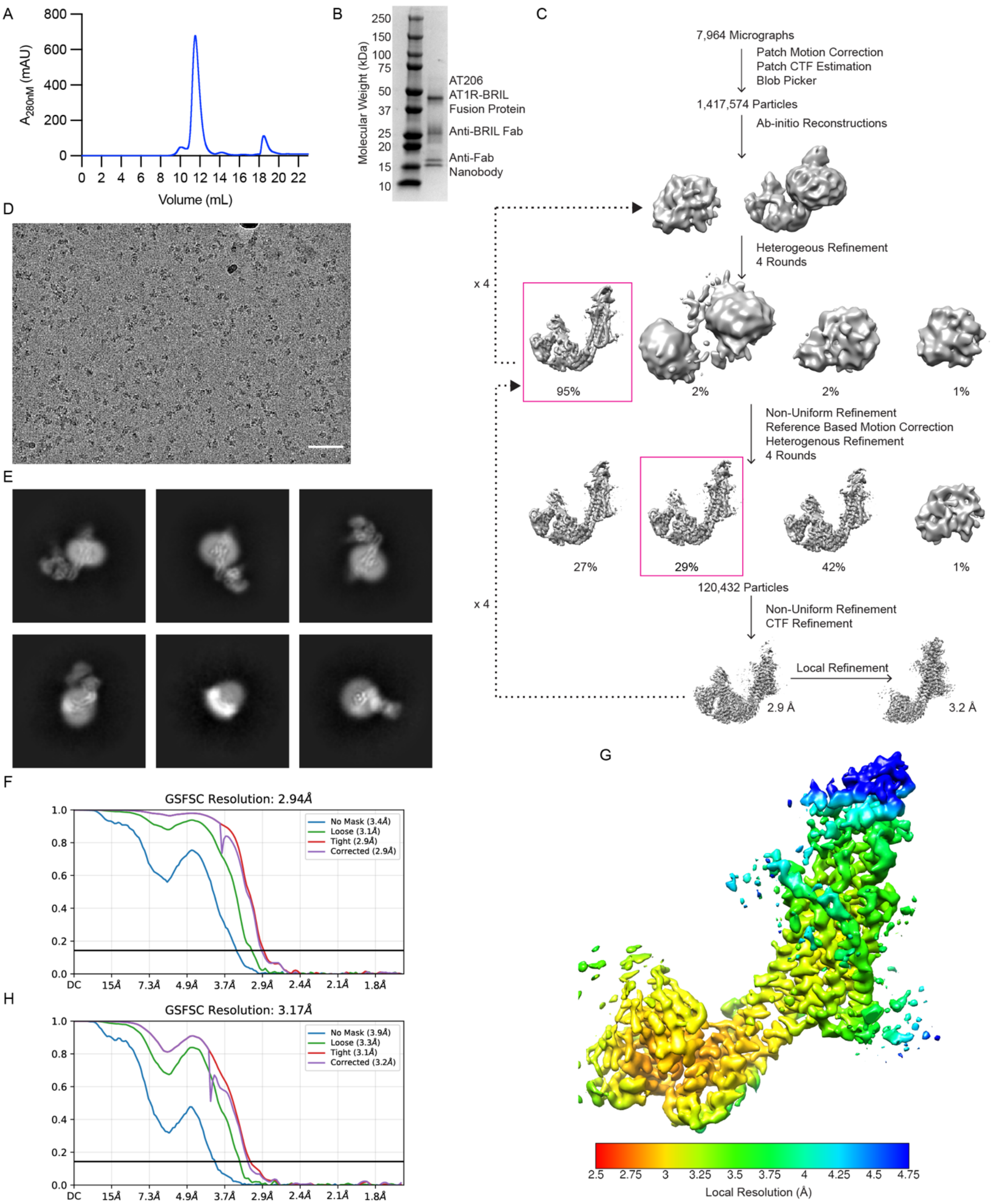

Fig. S10. AT206-AT1R data processing. A) Size exclusion trace, B) SDS-PAGE gel under reducing conditions, C) cryo-EM data processing scheme, D) representative micrograph (scale bar = 50 nm), and E) representative 2D class averages of AT206-AT1R-BRIL, anti-BRIL Fab, anti-Fab nanobody complex. F) Fourier shell correlation (FSC) used to determine global map resolution. G) Local resolution estimate of final map. H) FSC used to determine locally refined map resolution.

Table S1. Effects of AT110d4 Fusion on AngII Binding

	Log Ki (M)
FLAG-AT1R	-6.90 ± 0.03
FLAG-AT1R-AT110d4	-8.30 ± 0.12

Genetic fusion of AT110d4 to the C-terminus of AT1R enhances affinity of the purified receptor for AngII. Log Ki values represent the logarithm of the equilibrium dissociation constant and were calculated using the experimentally determined K_d value for [^{3}H]-Olmesartan; K_d FLAG-AT1R = 8 ± 0.6 nM, K_d FLAG-AT1R-AT110d4 = 6 ± 0.4 nM. Standard error was determined from three independent experiments.

Table S2. Nanobody Sequences

Nanobody	Sequence
AT201	QVQLQESGGGLVQAGGSLRLSCAAS**GTISDPPY**MGWYRQAPGKERELVA**AIDVGANT**YYADSVKGRFTISRDNAKNTVYLQMNSLKPEDTAVYYC**AVEYDAQVPHQY**WGQGTQVTVSS
AT203	QVQLQESGGLVQAGGSLRLSCAAS**GYISRSYD**MGWYRQAPGKERELVA**SINEGSTT**NYADSVKGRFTISRDNAKNTVYLQMNSLKPEDTAVYYC**AAYTYGHGDYHVY**WGQGTQVTVSS
AT205<br>AT214	QVQLQESGGGLVQAGGSLRLSCAAS**GYIFYDSV**MGWYRQAPGKEREFVA**AIAAGSST**NYADSVKGRFTISRDNAKNTVYLQMNSLKPEDTAVYYC**GEYYNWLHQY**WGQGTQVTVSS
AT206	QVQLQESGGGLVQAGGSLRLSCAAS**GSISYYR**MGWYRQAPGKEREFVA**GIGVGTTT**NYADSVKGRFTISRDNAKNTVYLQMNSLKPEDTAVYYC**AAYNYFPRSIVYYYVY**WGQGTQVTVSS
AT207	QVQLQESGGGLVQAGGSLRLSCAAS**GYIFTHSS**MGWYRQAPGKEREFVA**AITVGANT**NYADSVKGRFTISRDNAKNTVYLQMNSLKPEDTAVYYC**AAYNPFYPVYIY**WGQGTQVTVSS
AT208<br>AT215<br>AT216	QVQLQESGGGLVQAGGSLRLSCAAS**GYIFTRYR**MGWYRQAPGKEREFVA**AIGDGGTT**NYADSVKGRFTISRDNAKNTVYLQMNSLKPEDTAVYYC**AAYYVSRPNYGTIYVY**WGQGTQVTVSS
AT209	QVQLQESGGGLVQAGGSLRLSCAAS**GYIFYRYS**MGWYRQAPGKEREFVA**GISYGATT**NYADSVKGRFTISRDNAKNTVYLQMNSLKPEDTAVYYC**AAWGGRTLYFVY**WGQGTQVTVSS
AT210	QVQLQESGGGLVQAGGSLRLSCAAS**GTISSWSF**MGWYRQAPGKEREFVA**TIAPGSST**NYADSVKGRFTISRDNAKNTVYLQMNSLKPEDTAVYYC**AVYYGVPRFHVY**WGQGTQVTVSS
AT211	QVQLQESGGGLVQAGGSLRLSCAAS**GTISHSAG**MGWYRQAPGKERELVA**AISGGTIT**NYADSVKGRFTISRDNAKNTVYLQMNSLKPEDTAVYYC**AALGYWRRAGGTYYVVHYY**WGQGTQVTVSS
AT212	QVQLQESGGGLVQAGGSLRLSCAAS**GSISNYKY**MGWYRQAPGKERELVA**TIGGGATT**NYADSVKGRFTISRDNAKNTVYLQMNSLKPEDTAVYYC**AAVRAGYSLYYPYFYY**WGQGTQVTVSS
AT213	QVQLQESGGGLVQAGGSLRLSCAAS**GYIFYGAT**MGWYRQAPGKEREFVA**GINYGGST**NYADSVKGRFTISRDNAKNTVYLQMNSLKPEDTAVYYC**AVWDTYAQRRSSYFVY**WGQGTQVTVSS
AT118-<br>AT206	QVQLQESGGGLVQAGGSLRLSCAAS**DYIYRRYR**MGWYRQAPGKEREFVA**GIGVGTTT**NYADSVKGRFTISRDNAKNTVYLQMNSLKPEDTAVYYC**AAYNYFPRSIVYYYVY**WGQGTQVTVSS

CDR sequences are indicated in red

Table S3. Table of Nanobody K_d Values

	AT1R	AT1R + Losartan	AT1R + Olmesartan
AT206	ND	362.8 ± 26.7 nM	ND
AT118-H G26D CDR1, AT206 CDR 2&3	205.9 ±23.7 nM	152.6 ±18.9 nM	ND
AT209	ND	ND	102.2 ±17.2 nM
AT209 L103D	144.5 ±19.3 nM	ND	ND

K_d values for nanobodies binding to AT1R on the surface of Expi293F cells. Error represents the s.e.m. determined from three independent experiments. ND, not determined.

Table S4. Receptor Construct Sequences

Plasmid	Construct	Sequence
pMAS 85	*Signal Sequence*-FLAG-AT1R	*MKTIIALSYIFCLVFA*DYKDDDDKILNSSTEDGIKRIQDDCPKAGRHNYIFVMIPTLYSIIFV<br>VGIFGNSLVVIVIYFYMKLKTVASVFLLNLALADLCFLLTLPLWAVYTAMEYRWPFGNYL<br>CKIASASVSFNLYASVFLLTCLSIDRYLAIVHPMKSRLRRTMLVAKVTCIIIWLLAGLASLP<br>AIIHRNVFFIENTNITVCAFHYESQNSTLPIGLGLTKNILGFLFPFLIILTSYTLIWKALKKAY<br>EIQKNKPRNDDIFKIIMAIVLFFFFSWIPHQIFTFLDVLIQLGIIRDCRIADIVDTAMPITICIA<br>YFNNCLNPLFYGFLGKKFKRYFLQLLKYIPPKAKSHSNLSTKMSTLSYRPSDNVSSSTK<br>KPAPCFEVE
pMAS 183	*Signal Sequence*-AT118-H-linker-AT1RΔ320-linker-3C-proteinC	*MKTIIALSYIFCLVFA*EVQLVESGGGLVQPGGSLRLSCAASGYIYRRYRMGWYRQAPG<br>KGREFVAAISGGSSTNYADSVKGRFTISRDNSKNTVYLQMNSLRAEDTAVYYCAAYRI<br>VSDPRVYWGQGTQVTVSSLEGGSGGGSGILNSSTEDGIKRIQDDCPKAGRHNYIFVMI<br>PTLYSIIFVVGIFGNSLVVIVIYFYMKLKTVASVFLLNLALADLCFLLTLPLWAVYTAMEYR<br>WPFGNYLCKIASASVSFNLYASVFLLTCLSIDRYLAIVHPMKSRLRRTMLVAKVTCIIIWL<br>LAGLASLPAIIHRNVFFIENTNITVCAFHYESQNSTLPIGLGLTKNILGFLFPFLIILTSYTLI<br>WKALKKAYNIFEMLRIDEGLRLKIYKDTEGYYTIGIGHLLTKSPSLNAAKSELDKAIGRNT<br>NGVITKDEAEKLFNQDVDAAVRGILRNAKLKPVYDSLDAVRRAALINMVFQMGETGVA<br>GFTNSLRMLQQKRWDEAAVNLAKSRWYNQTPNRAKRVITTFRTGTWDAYEIQKNKPR<br>NDDIFKIIMAIVLFFFFSWIPHQIFTFLDVLIQLGIIRDCRIADIVDTAMPITICIAYFNNCLNP<br>LFYGFLGKKFKRYFLQLLKYGGSSLEVLFQGPEDQVDPRLIDGK
pMAS519	*Signal Sequence*-AT209-linker-proteinC-AT1R-226-BRIL-A2A/Frizzled5 H6-234-Δ320-linker-3C-Rho1D4	*MKTIIALSYIFCLVFA*QVQLQESGGGLVQAGGSLRLSCAASGYIFYRYSMGWYRQAPG<br>KEREFVAGISYGATTNYADSVKGRFTISRDNAKNTVYLQMNSLKPEDTAVYYCAAWG<br>GRTLYFVYWGQGTQVTVSSGGSGEDQVDPRLIDGKILNSSTEDGIKRIQDDCPKAGR<br>HNYIFVMIPTLYSIIFVVGIFGNSLVVIVIYFYMKLKTVASVFLLNLALADLCFLLTLPLWAV<br>YTAMEYRWPFGNYLCKIASASVSFNLYASVFLLTCLSIDRYLAIVHPMKSRLRRTMLVA<br>KVTCIIIWLLAGLASLPAIIHRNVFFIENTNITVCAFHYESQNSTLPIGLGLTKNILGFLFPFL<br>IILTSYTLIWKALKKAYDLEDNWETLNDNLKVIEKADNAAQVKDALTKMRAAALDAQKAT<br>PPKLEDKSPDSPEMKDFRHGFDILVGQIDDALKLANEGKVKEAQAAAEQLKTTRNAYI<br>QKYLERARSTLDKLNDDIFKIIMAIVLFFFFSWIPHQIFTFLDVLIQLGIIRDCRIADIVDTAM<br>PITICIAYFNNCLNPLFYGFLGKKFKRYFLQLLKYGGSSLEVLFQGPTETSQVAPA
pMAS528	*Signal Sequence*-AT206-linker-proteinC-AT1R-226-BRIL-A2A/Frizzled5 H6-234-Δ320-linker-3C-Rho1D4	*MKTIIALSYIFCLVFA*QVQLQESGGGLVQAGGSLRLSCAASGSISYYRMGWYRQAPGK<br>EREFVAGIGVGTTTNYADSVKGRFTISRDNAKNTVYLQMNSLKPEDTAVYYCAAYNYF<br>PRSIVYYYVYWGQGTQVTVSSGGSGEDQVDPRLIDGKILNSSTEDGIKRIQDDCPKAG<br>RHNYIFVMIPTLYSIIFVVGIFGNSLVVIVIYFYMKLKTVASVFLLNLALADLCFLLTLPLWA<br>VYTAMEYRWPFGNYLCKIASASVSFNLYASVFLLTCLSIDRYLAIVHPMKSRLRRTMLV<br>AKVTCIIIWLLAGLASLPAIIHRNVFFIENTNITVCAFHYESQNSTLPIGLGLTKNILGFLFP<br>FLIILTSYTLIWKALKKAYDLEDNWETLNDNLKVIEKADNAAQVKDALTKMRAAALDAQK<br>ATPPKLEDKSPDSPEMKDFRHGFDILVGQIDDALKLANEGKVKEAQAAAEQLKTTRNA<br>YIQKYLERARSTLDKLNDDIFKIIMAIVLFFFFSWIPHQIFTFLDVLIQLGIIRDCRIADIVDT<br>AMPITICIAYFNNCLNPLFYGFLGKKFKRYFLQLLKYGGSSLEVLFQGPTETSQVAPA

Table S5. Cryo-EM data collection, refinement and validation statistics

	AT209 AT1R Olmesartan (EMDB-47831) (PDB 9EAH)	AT206 AT1R Losartan (EMDB-47832) (PDB 9EAI)	AT206 AT1R (EMDB-47833) (PDB 9EAJ)
**Data collection and processing**			
Magnification	105,000	105,000	105,000
Voltage (kV)	300	300	300
Electron exposure (e–/Å^2)	64.1	64	63.5
Defocus range (μm)	-0.8 to -1.8	-0.8 to -1.8	-0.8 to -1.8
Pixel size (Å)	0.83	0.83	0.83
Symmetry imposed	C1	C1	C1
Initial particle images (no.)	903,168	1,748,565	1,417,574
Final particle images (no.)	251,356	379,250	120,432
Map resolution (Å)<br>FSC threshold	3.0<br>0.143	3.1<br>0.143	2.9<br>0.143
**Refinement**			
Model resolution (Å)	3.1	3.1	3.2
Model composition<br>Non-hydrogen atoms<br>Protein residues<br>Ligands	 5606<br>702<br>2	 5554<br>687<br>4	 5426<br>662<br>0
R.m.s. deviations<br>Bond lengths (Å)<br>Bond angles (°)	 0.002<br>0.44	 0.003<br>0.48	 0.004<br>0.51
Validation<br>MolProbity score<br>Clashscore<br>Poor rotamers (%)	 1.55<br>5.63<br>0.68	 1.47<br>6.26<br>1.37	 1.9<br>7.55<br>1.78
Ramachandran plot<br>Favored (%)<br>Allowed (%)<br>Disallowed (%)	 96.4<br>3.6<br>0	 97.9<br>2.1<br>0	 95.8<br>4.2<br>0
